# The right way to ride the wrong bike: An exploration of Klein’s ‘unridable’ bicycle

**DOI:** 10.1371/journal.pone.0315769

**Published:** 2025-01-02

**Authors:** B. D. Coller

**Affiliations:** Department of Mechanical Engineering, Northern Illinois University, DeKalb, Illinois, United States of America; Federal University of Technology - Parana, BRAZIL

## Abstract

Professor Richard Klein and his students built a bicycle with a rather interesting feature: no one was able to ride it. A prize was offered. Hundreds of students and cycling enthusiasts attempted it. Years passed, and the prize money grew. Klein’s rear-steered bicycle became a canonical example of how non-minimum phase systems can be difficult and sometimes nearly impossible to control. It has been lauded as a particularly effective educational example in which students can *experience* the loss of controllability in a seemingly simple, albeit unorthodox bicycle. The primary result of the work reported here is a demonstration that it is possible for a human of modest athletic ability to ride Klein’s unridable bicycle, to keep it balanced, and to control its direction of travel. There is a secret to riding Klein’s rear-steer bicycle. The secret is revealed through an exploration of the dynamics and control of the bike that contains three elements: (1) modeling the physics of the actively steered bicycle as an inverted pendulum riding atop a carriage; (2) recognizing that the steer kinematics leads to competing physical mechanisms which an aspiring rider might exploit; and (3) examining limitations of controllability and stabilizability of the system from a state space perspective. From this vantage point, one can devise a novel strategy, based on a component of lateral acceleration that dominates at low speed, for riding the so-called “unridable” bike and solving Klein’s puzzle. The work adds a new chapter on the dynamics and control of the rear-steered bicycle, a problem of academic interest.

## Introduction & background

**Fig 1:**
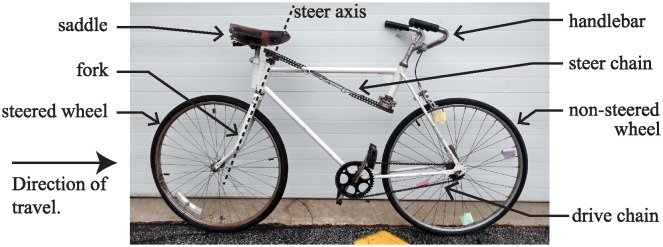
Klein’s “unridable bicycle”. A photograph of the rear-steered bicycle that Richard Klein and others deemed unridable.

In 1986, Richard Klein and his students made an unusual modification to a rather normal bicycle [1, 2]. A photograph of the bicycle is shown in Fig 1. They placed the handlebar where the seat normally goes and moved the seat to where the handlebar normally belongs. They also moved the chain ring and wheel drive sprocket to the other side of the bike. Therefore, when a rider sits on the seat, places their hands on the handlebar in front of them, and begins pedaling, the bicycle begins moving in the direction that the rider is facing, as expected. What makes the bicycle different is that the wheel which steers the bike is the rear wheel. The unsteered wheel, which is now at the front of the bicycle, is mechanically connected to the pedals via drive chain. Klein gave a name to this bike: Rear-Steered Bike I (RSB1).

**Fig 2:**
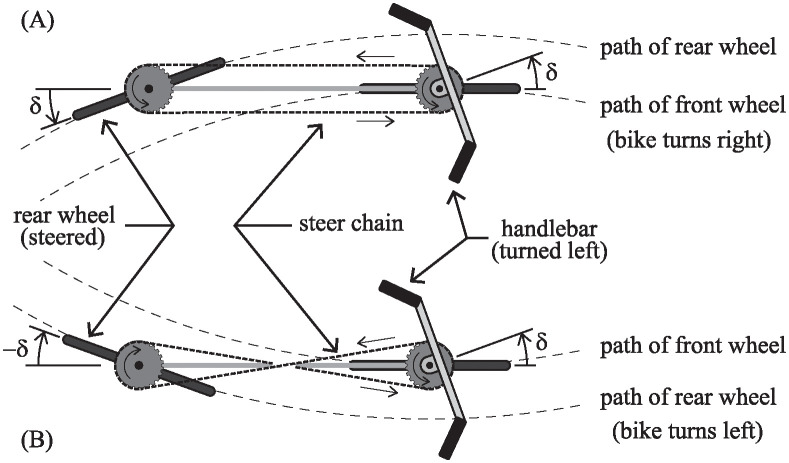
Steer chain configuration. A: When the steer chair wraps around the sprockets in a simple loop, a leftward turn of the handlebar causes the rear-steered bicycle to execute a rightward turn. B: When the steer chain is in a figure eight configuration, the bicycle turns in the same direction that the handlebar is turned.

For Klein’s bicycle shown in Fig 1, the handlebar and fork are separated from each other and placed at opposite ends of the bike as shown in the photo of Fig 1. The two parts are connected to each other mechanically via the steer chain wrapped around two steer sprockets of equal diameter. If the steer chain is wrapped in a simple loop configuration as shown in Fig 2A, then a left turn of the handlebar by angle *δ* causes the fork and the steered wheel to rotate the same angle *δ* and in the same direction. However, since the steered wheel is at the rear of the bicycle, such a leftward turn of the handlebar causes the entire bike to turn to the right.

Another way to configure the steer chain is shown in Fig 2B. Here, the chain makes a figure eight shaped loop. As a result, the fork turns in opposite direction as the handlebar. In this case, the entire bicycle turns in the same direction that the handlebar is rotated. The rider may choose either configuration on Rear-Steered Bike I (RSB1).

From a control theory perspective, it does not matter which way the steer chain is configured. If it is possible to keep the bicycle balanced with the chain in one configuration, then, in theory, it should be possible to stabilize the bike with the exact same control strategy, except for turning the handlebar in the opposite direction. The only problem is that humans do not operate as symmetrically as bicycles do. Therefore, the purpose of being able to reconfigure the chain, as illustrated in Fig 2, is for the convenience of riders to choose what is most comfortable for themselves.

Regardless of which way the steer chain is configured, the simple act of making the rear wheel the one that steers, while keeping the center of mass of the bike close to the rear wheel, is what makes the bicycle exhibit odd behavior, and makes it exceedingly difficult to ride. In the Supporting Information section at the end of this article, there are links to several videos. The first, S1 Video. shows engineering students attempting to ride Klein’s Rear-Steered Bike I. To be fair the video shows students’ first interactions with the bike. However, it typically does not get much better after spending hours trying to wrap their minds around it.

Gordon et al. [3] provide a general discussion on the dynamics of rear-steered bicycles. De Jong [4] has documented an extensive history of of rear-steered bicycles going back more than a century. Some rear-steered bikes are very ridable, even ridable without hands (self-stable). Others, including Klein’s RSB1, have been reported to be difficult if not impossible to ride.

### Klein’s challenge

Richard Klein, a tinkerer and scholar inspired by the work of Jones [5], made a career out of exploring the kinematics and dynamics of bicycles. Klein would reconfigure bicycles in ways that made it possible for people with disabilities to experience the joy and sense of accomplishment from riding a bike [6, 7]. As an educator, Klein built a large collection of oddly-configured bicycles that serve as tangible examples of difficult theoretical concepts that engineering students encounter in system dynamics and control courses [1, 8]. In this effort, Klein’s Rear-Steered Bike I (RSB1) became the canonical example of the perils one can encounter when trying to control an unstable non-minimum phase system, a topic that we will return to in this article. The rear-steered bike was the highlight of a 2005 *IEEE Control Systems Magazine* article co-written with Åström, and Lennartsson [2].

Richard Klein openly challenged the public to try to ride his “unridable” Rear-Steered Bike I (RSB1). He broadcast that challenge to the control systems community in the 2005 IEEE article. At that time, the prize money for the first person to successfully ride the bike was $1,000 US. By 2006, the prize had risen to $5,000 US [9].

Recognizing the potential difficulty posed by a bicycle that steers in the opposite direction that the handlebars are pointed, Klein allowed those seeking conquer the “unridable” bike to choose which configuration from Fig 2 that they wanted for the steer chain. To successfully complete Klein’s challenge, the rider had to remain seated in the saddle, with both feet on the pedals. Furthermore, both wheels had to remain contact with the ground at all times, and the bike needed to make continuous forward progress along the specified path. The path started with a straight section of 100 ft (30.5 meters) in length, followed by a 90-degree turn, followed by a second 100 ft long straight section. At all times, the bicycle had to remain a distance of 3 ft (0.9 meters) from the center of the path. When the rider reached the end of the path, they were allowed to put their feet on the ground to manually turn the bike around. Then, the rider had to ride again, this time traversing the path in the opposite direction.

In an archived web postings [9, 10], Klein stated that over the years, many hundreds of overly optimistic riders attempted to win the prize reward. For some of the most skilled bicyclists and unicyclists, Klein would let them borrow the bike for a month or more to develop their skills. Before the autumn of 2009, there was only one person who was able to remain upright. However, this rider ended up riding haphazardly in an open and flat parking lot as opposed to being able to follow a prescribed path, a necessary condition for winning the prize.

### Klein’s bike differs from a “Backward Brain Bicycle”

It is well known that the simple act of switching the sign of the input/output relationship, so that a bike turns in the opposite direction that the handlebar is steered, is sufficient to make the task of balancing a bicycle upright extraordinarily difficult. The popular YouTube channel, *Smarter Every Day*, has an episode devoted to this specific topic [11] in which Destin Sandlin added a geared connection between the handlebar and fork of a normal front-steered bicycle. When the rider would turn the handlebar one way, the fork would turn the front wheel by the same angle about its steer axis, but in the opposite direction.

To be clear, Sandlin and co-workers did nothing else to modify the dynamics of their “Backwards Brain Bicycle.” From a control theory perspective in which the input to the bicycle system is the rider’s steer action, and the output is the lean angle, the difference between a normal bicycle and Sandlin’s Backward Brain Bicycle is a trivial sign change. Theoretically, both bicycles are equally controllable and equally stabilizable. The rider would only need to provide the opposite steer input in response to a perceived amount of bicycle lean.

In practice, however, the Backwards Brain Bicycle is difficult to ride because of the *human* implementing the control strategy. In Sandlin’s case, it took eight months of daily dedicated training to de-program the subconscious part of the brain that holds automatic responses to perceived lean, and replace those responses with ones that produce the opposite handlebar motion.

The story of the “Backward Brain Bicycle” is relevant here in that it illustrates the minor similarities, and the major differences between it and Klein’s “Rear-Steered Bike I” (RSB1). Switching direction of the steer action (with a “Backward Brain” gear or with a reconfigurable steer chain loop in Fig 2) does *not* theoretically make a bicycle more controllable or less controllable. It just changes the rider’s perspective, making balancing the bike more difficult or less difficult depending on how the rider’s mind is trained to respond to a leaning bicycle. To accommodate different riders’ preferences, Klein allowed either steer chain configuration.

However, that is where the similarities end. Klein’s RSB1 is a rear-steered bicycle, and the Backward Brain bike is a front-steered bicycle. As illustrated later in this article, front-steer and rear-steer bikes are fundamentally different in how the steer/lean interactions work. For a rear-steered bike, there are situations where it is essentially impossible to keep it balanced with steer input alone. Understanding how this works might lead to developing a strategy for solving Klein’s puzzle.

### Blame it on the open loop zero

To provide a control theoretic explanation of why Klein’s RSB1 is “unridable”, Åström, Klein, and Lennartsson [2] derived linearized equations of motion for a simplified bicycle model and constructed transfer functions that relate the lean angle of the bike (output) to the rider torque on the handlebar (input). In an alternate formulation [12], the input is the steering angle *δ*. They observed that the system has two open loop poles, both real, one negative and the other positive. The two open-loop poles come from the fact that the bicycle behaves like an inverted pendulum, tending to fall toward one side or the other. The transfer function also had an open loop zero. For a bicycle with front wheel steering, the zero resides on the negative real axis, making it fairly straightforward to devise control laws which could stabilize the system.

However, in the case of a rear-steered bicycle, the open-loop zero is on the positive real axis, making it a non-minimum phase system. Furthermore, for system parameters approximating those of Klein’s unridable bike, the open loop zero and the unstable open loop pole resided in close proximity over a wide range of bike speeds. Such non-minimum systems are often impacted by fundamental limitations on robustness.

In a general treatise on *Limitations of control system performance*, Åström [12] used the rear-steered bicycle as a canonical example of a SISO system with poles and zeros in the right half plane. Here, he estimated the upper bound for the phase margin to be about 10^o^. This indicates that a rear-steered bicycle, similar to Klein’s unridable bike, might be stabilizable in principle. However, such a controller would lack an appropriate level of robustness for a feedback controller that consists of the human brain serving as the real-time signal filter and control law processor, and the human muscles serving as actuators. For robustness, Åström [12] recommends zero-to-pole ratios that are either less than 0.25 or greater than 4.0.

To achieve zero-to-pole ratios in this robustness range, Åström [13] and Suryanarayanan et al [14] suggests riding strategies that would violate the rules of Klein’s challenge.

### Scope of the current work

The author’s interest in this particular bicycle dynamics and control problem is multi-dimensional. First, from an educational perspective, the author fully agrees with the arguments posed in [1, 2, 13] that the bicycle is a compelling platform to teach system dynamics and control. Students can do real-world mathematical modeling of a tangible system, witness stability/instability, make connections to the mathematics, and realize the power of feedback control, and experience the loss of controllability.

Secondly, the students and the author have shared a certain degree of dissatisfaction with the claim that Klein’s RSB1 is impossible to ride. Yes, the arguments made in [2, 12, 13] seem rational and sound. But to many mechanical engineers, arguments made with transfer functions in the Laplace domain seem to obscure what is physically happening with the mechanical system, in the real world, which is subject to forces, mechanical constraints, and inertial effects. What, physically, makes a rear-steered bike so different than a front-steered bike? What does non-minimum phase mean, physically, in the context of the bicycle problem? How does the coincidence of the non-minimum phase behavior with the unstable part of the dynamics yield a system that is exceedingly difficult to control? And finally, the big question: *Can a more physical understanding of what makes Klein’s rear-steered bike so difficult to ride yield insight into how to exploit the physics and solve the puzzle?*

One of the contributions of this work is a re-telling, and re-exploration of Klein’s unridable bike from a mechanical, time domain, state space perspective that allows mechanically inclined students and researchers to understand the questions posed above. This consists of three components:

1. **Modeling the dynamics of the bicycle.** This is more than just generating equations of motion; it is about thinking of the bicycle as an inverted pendulum that is actuated by lateral acceleration of the base generated by steering action.
2. **Steer Kinematics.** Recognizing that the act of steering the bike generates two different types of lateral acceleration. One is a centripetal acceleration proportional to the speed of the bike, squared. The other is a “swing” acceleration linearly proportional to bike speed. For a traditional front-steered bike, the two components of acceleration reinforce each other, making it unnecessary to distinguish them. However, for the rear-steered bicycle, the two acceleration components generally work against each other, giving rise to the “non-minimum phase” behavior.
3. **Controllability and Stabilizability.** The equations of motion are linearized and cast into state space form as a system of first order differential equations. Investigating, geometrically, how the control vector field coming from the steer input aligns (or doesn’t align) with the natural uncontrolled dynamics of the bike, one can investigate fundamental limitations and opportunities for control and stabilization.

The result of this exploration yields an answer to the “big question” mentioned previously. By the end of this article, the author demonstrates that, in fact, it is possible for a human of modest athletic ability to ride Klein’s “unridable” bike. The solution to the puzzle is not elegant to watch, but it is an interesting exploitation of the swing component of acceleration at low bike speeds. It is a result of academic interest and educational value.

## Bicycle models

**Fig 3:**
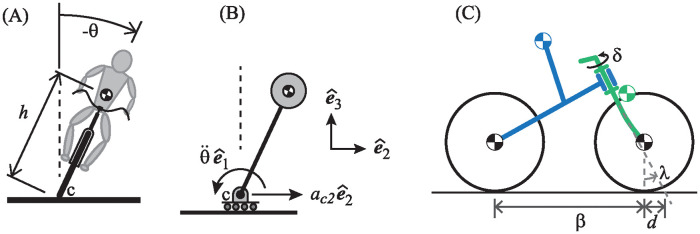
Bicycle modeling. (A) Modeling the lean dynamics of a bicycle as an inverted pendulum. (B) To balance the pendulum, one must accelerate the base in the direction of the lean. (C) A Carvallo-Whipple-type bicycle model.

There is a long history of developing mathematical models for bicycles and motorcycles [15–17], going back to the ground-breaking work of Carvallo [18] and Whipple [19] at the end of the 19^th^ century. The foundation behind all these models is that a bicycle or motorcycle is essentially an inverted pendulum [3] as depicted in Fig 3A. A stationary bicycle, for example, will simply fall over if not supported by a kickstand, or by other means.

Anyone who has had the experience of balancing a broomstick in the palm of one’s hand knows that the secret to keeping the pendulum upright is to pay careful attention to the direction in which it is leaning, and then to sufficiently *accelerate* the base of the pendulum into the direction of the lean. To balance a bicycle, normally the rider must first get it moving, and then *the bike must steer into the direction of the lean to generate sufficient lateral acceleration to prevent it from falling over.* This steering action into the direction of the lean can occur via direct effort by the rider to turn the handlebar, or it could occur through an unassisted physical interaction between the lean dynamics and the steer dynamics of the bike (e.g. hands-free riding).

To develop an understanding of how the steer kinematics of a rear-steered bike generates acceleration to affect the lean dynamics, this study utilizes two different types of models. The first is a *simplified model* that strips away all but the essential elements needed to illuminate what makes Klein’s RSB1 so difficult, if not impossible, to ride. The second type of model is a *validation model* whose purpose is to ensure that the simplified model is not over-simplified.

### Validation model

Carvallo [18] and Whipple [19], in effort to capture the lean/steer dynamics, modeled the bicycle as four inter-connected rigid bodies: two wheels, a frame with a mass representing the rider rigidly attached to it, and a steer assembly consisting of the handlebar and fork which the steered wheel is attached to. The Carvallo-Whipple model of the bike is depicted in Fig 3C. If the bike represented in the figure rolls to the right, it is the front wheel that steers the bicycle. One obtains a rear-steered bike model simply by reversing the direction of its velocity.

Deriving error-free equations of motion is a substantial task. Meijaard et al. [15] provide a detailed history of efforts to do so, spanning more than a century. In their accompanying paper [20], the authors published what they claim to be an error-free system of differential equations that contains 25 mass and configuration parameters, including a set of typical parameters for a “benchmark bicycle”.

In this bicycle model, all four bodies have a side-to-side reflection symmetry. Other than that, and the fact that the centers of mass of the wheels lie at the center of the circular bodies, the mass distribution of the bodies is arbitrary. The wheels are assumed to have knife-edge, non-slip, rolling contact with a flat ground. The wheel bearings and steer hinge are assumed to be friction-free.

When the bicycle is upright, the steer axis generally makes an angle λ relative to vertical, creating a mechanical trail or caster for the rear wheel as indicated by the symbol *d* in Fig 3C.

The ways in which leaned and steered bicycle wheels can contact the ground make full nonlinear modeling wheel kinematics complicated [21]. This is one of the reasons bicycle models are linearized at the outset. At linear order, the longitudinal dynamics (speed up/slow down) of the bike decouple from the from the lateral (lean/steer) dynamics. For this reason, one typically sets a constant speed, *v*, at which unsteered wheel of the bike travels.

The end result of this modeling effort by Meijaard et al is a set of two coupled, second-order, linear differential equations for the lean angle *θ* and steer angle *δ* which take the following form:

$$\begin{array}{c} \left[\begin{array}{cc} M_{\theta \theta } & M_{\theta \delta }\\ M_{\delta \theta } & M_{\delta \delta } \end{array}][\begin{array}{c} \ddot{\theta }\\ \ddot{\delta } \end{array}]+v[\begin{array}{cc} 0 & C_{1\theta \delta }\\ C_{1\delta \theta } & C_{1\delta \delta } \end{array}][\begin{array}{c} \dot{\theta }\\ \dot{\delta } \end{array}\right]\\ +[g[\begin{array}{cc} K_{0\theta \theta } & K_{0\theta \delta }\\ K_{0\delta \theta } & K_{0\delta \delta } \end{array}]+v^{2}[\begin{array}{cc} 0 & K_{2\theta \delta }\\ 0 & K_{2\delta \delta } \end{array}]][\begin{array}{c} \theta \\ \delta \end{array}]=[\begin{array}{c} 0\\ \tau \end{array}]. \end{array}$$ (1)

Here, *g* = 9.81m/s^2^ is the gravitational field strength and coefficients *M*_*xx*_, *C*_1*xx*_, *K*_0*xx*_, and *K*_2*xx*_ depend on the 25 mass and configuration parameters mentioned previously. Symbol *τ* represents a steer torque input from the rider in effort to turn the handlebar. In the original formulation of this model, there is also an external torque input on the frame corresponding to the rider leaning relative to the frame. However, Kooijman et al. [22] have observed that human riders stabilize bicycles primarily by steering control actions with very little upper body lean. The finding is consistent with those of Sharp [23].

The model given by Eq (1) are particularly useful in the study of bicycle self-stability. One can think of this as a dynamic coupling between lean and steer that causes the bike to naturally steer into the direction it is leaning. This coupling can provide a self-stability that allows the bike/rider to remain balanced without their hands on the handlebar. To perform such a self-stability analysis, one would set the steer torque to zero, *τ* = 0, and then calculate the eigenvalues of the system of differential equations over a range of speeds. Typical front-steered bicycles often have a range of speeds for which they are self-stable. Typical bicycles are generally not self-stable at any speed when they are operated in reverse, making them rear-steered bikes.

Performing self-stability analyses and then comparing the behavior to that of a well-instrumented physical bicycle is also a fruitful approach for validating the mathematical model. For example, Kooijman et al. [24] show that the Meijaard et al. model (Eq (1)) is capable of predicting the growth rate and frequency of the weave mode of an an actual bicycle for which the physical parameters have been painstakingly measured. The close match, they argue, suggests that tire slip, tire compliance, and frame/fork compliance are not important effects at speeds less than 6 m/s.

In another study that effectively validated the model, Kooijiman et al. [25] used the Meijaard et al. model to find a narrow range within the high dimensional parameter space for which it is possible to design a bicycle without gyroscopic effects, and without caster effects, that can still be self-stable. Then they built the bike and proved that the model’s predictions were correct.

Similarly, deJong [4] developed an automated search through the 25 parameters of the Meijaard et al. model, to find and example of a rear-steered bicycle, very different from those of RSB1, is self-stable. Again, upon building a bike that matched the parametric description, the behavior of the physical bike matched that of the mathematical model.

### Steer angle control

Since the Klein’s RSB1 is not self-stable, the goal here is to seek active feedback control strategies that a human rider can implement. The strategies would need to balance (or stabilize) the bike with sufficient ease that the rider can also direct the bike along a desired path. As described by Schwab and Meijaard [26], it is possible to formulate a bicycle control problem as one for which the steer torque, *τ*, is the control input, or one can treat the steer angle, *δ*, is the input. Since the steer axis degree of freedom for the bike has relatively little rotational inertia, and since the bike studied here will be moving at speeds which gyroscopic and caster effects will be small, it is relatively easy for a rider of modest strength to rapidly turn the handlebar to any desired angle on time scales significantly small compared to time scales of the falling bike/pendulum.

When one formulates the control problem as one for which the steer angle is specified directly by the rider/controller, rather than the result of a Newtonian dynamic response to torque input, the bike model becomes simpler. The bottom second order equation in (1) is removed, yielding

$$\begin{array}{c} M_{\theta \theta }\;\ddot{\theta }+g\;K_{0\theta \theta }\;\theta =-M_{\theta \delta }\;\ddot{\delta }-g\;K_{0\theta \delta }\;\delta -v\;C_{1\theta \delta }\;\dot{\delta }-v^{2}\;K_{2\theta \delta }\;\delta . \end{array}$$ (2)

This is the “validation model” for the current study. It is a validated linearized model that correctly captures the physics of the bicycle’s lean in response to steer angle input. Note that the terms on the left side of Eq (2), provided that coefficient *K*_*θθ*_ is negative, are the same as those of the linearized inverted pendulum. As we develop an even simpler model for the bike in the next subsection, the validation model will help quantify and justify the simplifying assumptions to be made.

### Elements of a simplified model

Normally, in model-based control, once one has a mathematical model like that of Eq (2), they may move onto the next step and start devising controllers. However, in this case of finding a strategy for a human to ride the bicycle, the approach here might need to be different. In this case, the controller must be decipherable by a human rather than programmed into a computer. Since a human must ride the bike, a human must be able to process the control algorithm. And for this particular human taking on the challenge, the author, a mechanical engineer, it is helpful to have a *physical* understanding of what makes the bike so difficult to ride. It would be helpful to be able to pinpoint some primary physical interactions, that a rider could *feel, interpret, and act upon* in real time in effort to keep the bike balanced.

The next step, therefore, is to simplify the bicycle model more, removing small, relatively unimportant effects, in effort to expose key aspects of the dynamics that can be exploited for control of the bike. These simplifications take the form of assumptions to be validated later:

- **Assumption A1: One can ignore gyroscopic effects.** For a front-steered bicycle, it is well-known that the gyroscopic effect coming from rotating wheels can play an important role in bike self-stability. When gravity pulls a leaning bike downward, it causes a moment on the front rotating wheel. The gyroscopic effect causes that wheel to rotate about the steer axis, turning into the lean. Such a turn subsequently causes a lateral acceleration to upright the bike. This gyroscopic coupling between the lean and steer dynamics is a self-stabilizing mechanism. Jones [5] and Klein [1, 2] reported on experiments in which they added a counter-rotating wheel, in contact with the steered wheel, but not touching the ground. This extra wheel canceled the angular momentum of the steered wheel, thus removing the gyroscopic effect. As a consequence, these particular front-steered bicycles were no longer self-stable. However, when humans attempted to ride gyro-less bicycles, they did so with ease. Human riders do not need gyroscopic coupling to rid a bike; they can take control of the bike and balance it manually. To ride RSB1, a human rider is going to have to take control of the bike and manually override any gyroscopic tendencies. Since the bike will be traveling at low speed, there is reason to believe that any gyroscopic effects will be small and will not hinder the rider’s ability to steer the handlebar in a desired manner.
- **Assumption A2: One can ignore caster effects and those of a tilted steer axis.** The bicycle diagram in Fig 3C shows typical geometry of a front-steered bike where the point at which the front (steered) wheel contacts the ground lies behind the point where the steer axis passes through the ground. The offset, labeled *d* in the figure, causes a self-aligning caster effect, much like the caster wheels on a shopping cart or office chair. As with the gyroscopic effect, this caster offset can have some self-stabilizing effects. Also, like the gyro, Jones [5] a human rider can stabilize a bike, without much extra effort, whether the bike has positive, negative, or zero caster.
  Since experimental evidence seems to indicate that the caster effect has small impact on balanceability of a front-steered bicycle, under direct control of a rider, we remove it as an effect to explore in the ridability of a rear-steered bike. To remove the caster, this study explores a bicycle model for which the fork is straight and steer axis is vertical when the bike has zero lean. In this configuration, the contact point between the wheel and the ground lies on the vertical steer axis.
- **Assumption A3: One can neglect the effect of the center of mass of the steering assembly not lying on the steer axis.** The previous simplification removed the geometric caster. If one could also require that the center of mass of the steer assembly lies on the steer axis then, it would also remove an inertial caster effect. Interestingly, Kooijman [25] showed that it is possible to design a self-stable bike with cancelling gyroscopic effects and zero geometric caster that can exhibit self-stability with this inertial offset alone.
  Just like the other caster effect, it seems reasonable to expect a human rider providing steer angle input to be minimally effected by this inertial offset. With this and the previous simplifications, the coefficients *M*_*θδ*_ and *K*_0*θδ*_ in Eq (2) both go to zero, simplifying the way that steer angle input *δ* effects the lean dynamics.
- **Assumption A4: One can simplify the mass distribution in the models.** In the validation model, the mass distribution of the frame and the steering assembly bodies were arbitrary, apart from the lateral side-to-side symmetry. To simplify the model in this study, the lean dynamics of the bike will be treated as a simple pendulum parameterized by the distance of its center of mass above the lean axis and a single rotational moment of inertia of the pendulum about that lean axis.
  Simplifying the mass distribution in a bicycle model does have precedent. For example, in the attempt of Kooijman et al. [25] to find a self-stable bike without gyroscopic or caster effects, the researchers resorted to a simple two point-mass model for the bike. Even with the simplest non-gyroscopic, caster-less, possible mass model that one could devise, they were able to find a favorable combination of parameters that would work. The researchers designed an actual “bike,” with distributed mass throughout, that exhibited the hypothesized self-stability mechanism.

In S1 Appendix, the validation model is used to test these simplifying choices.

### Mechanical equivalent to the simplified model

All mathematical models of the bicycle, going back more than a century, treat the bike as an inverted pendulum. Balancing the bicycle is achieved by accelerating the base of the pendulum laterally, into the direction of the fall. By removing or ignoring certain elements of the bike model as outlined above, one is effectively removing or constraining certain mechanical aspects of the bike.

**Fig 4:**
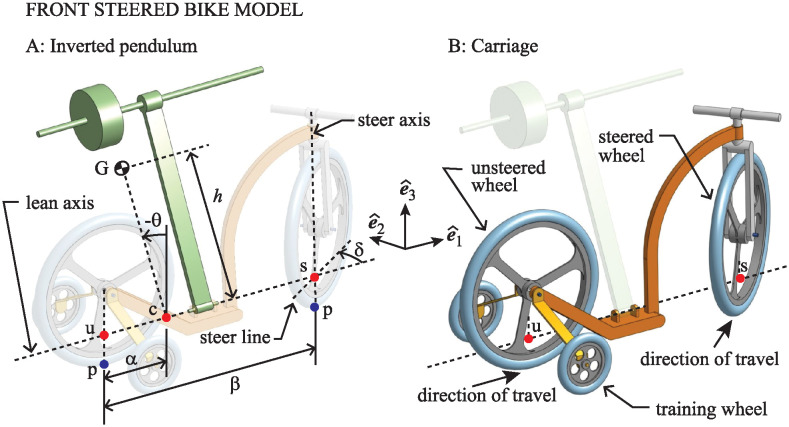
Mechanical equivalent of the simplified model of the front-steered bicycle. The model consists of an inverted pendulum attached to a carriage simplifying the steer kinematics and its interaction with the lean dynamics.

For the front-steered bike, the simplified bicycle model becomes mechanically equivalent to that depicted in Fig 4. It consists of a pendulum that is free to swing side to side around a lean axis that is affixed to a carriage. The carriage consists of a sing-track front/rear pair of wheels to produce the appropriate steer kinematics, but equipped with training wheels that keep the wheels upright so that ground contact geometry is as simple as possible. The steer axis is vertical, with a straight fork and zero caster. This quashes any gyroscopic effect and isolates the steer kinetics completely from the lean degrees of freedom of the pendulum.

In accordance with the way in which longitudinal and lateral dynamics of the bicycle decouple at linear order [26], the center of the unsteered wheel is assumed to move at constant speed *v*. If the direction of travel is as indicated in Fig 4, then one has a simplified mechanical model of a front-steered bicycle. If one were to change the sign of *v*, reversing the direction of travel, then one obtains a simplified model of the rear-steered bike.

**Fig 5:**
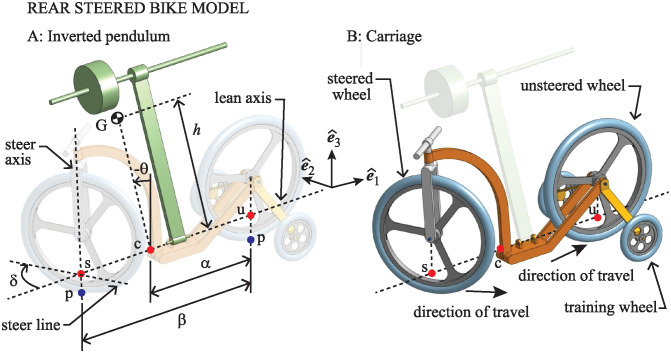
Rear-steered version of the simplified mechanical model of the bicycle.

The actual rear-steered bike model we use in this study is depicted in Fig 5. Angles are redefined so that a negative *θ* corresponds to a lean to the left and a positive *δ* corresponds to a steer to the left in the same way that they do for the front-steered bicycle.

### Simplified mathematical model: Lean dynamics

Figs 4 and 5 show a coordinate frame labeled $\left({\hat{\text{e}}}_{1},{\hat{\text{e}}}_{2},{\hat{\text{e}}}_{3}\right)$ fixed to the moving carriage. For both the front-steered and rear-steered bike models, basis vector ${\hat{\text{e}}}_{1}$ is parallel to the lean axis of the pendulum, pointed in the direction of motion. Vector ${\hat{\text{e}}}_{3}$ is vertically upward, and ${\hat{\text{e}}}_{2}$ is lateral, positive to the left. As the pendulum swings, its center of mass, G, moves in a vertical plane in the $\left({\hat{\text{e}}}_{1},{\hat{\text{e}}}_{2},{\hat{\text{e}}}_{3}\right)$ frame, perpendicular to the lean axis. That plane intersects the lean axis at point c labeled in the two figures.

A careful derivation of the equations of motion for the pendulum accounts for the fact that point c can accelerate in the horizontal plane: ${\vec{a}}_{c}=a_{c1}{\hat{e}}_{1}+a_{c2}{\hat{e}}_{2}$. Also, the $\left({\hat{\text{e}}}_{1},{\hat{\text{e}}}_{2},{\hat{\text{e}}}_{3}\right)$ frame fixed to the carriage can rotate about the ${\hat{\text{e}}}_{3}$ axis with a yaw rate of $\dot{\psi }$. Doing so yields

$$\begin{array}{c} I_{c1}\ddot{\theta }=mgh\;\text{sin}\left(\theta \right)+mh\;a_{c2}\;\text{cos}\left(\theta \right)+\left(I_{c2}-I_{c3}\right){\dot{\psi }}^{2}\;\text{cos}\left(\theta \right)\;\text{sin}\left(\theta \right). \end{array}$$ (3)

Here, *m* is the mass of the pendulum, *h* is distance of the center of mass from point c, and *g* is the gravitational field strength. *I*_*c*1_, *I*_*c*2_, and *I*_*c*3_ are moments of inertia about point c corresponding to directions ${\hat{\text{b}}}_{1}$, ${\hat{\text{b}}}_{2}$, and ${\hat{\text{b}}}_{3}$ aligned with principal axes of the bike/rider. Directions $\left({\hat{\text{b}}}_{1},{\hat{\text{b}}}_{2},{\hat{\text{b}}}_{3}\right)$ align with $\left({\hat{\text{e}}}_{1},{\hat{\text{e}}}_{2},{\hat{\text{e}}}_{3}\right)$ when *θ* = 0.

The result is similar to the bicycle model derived by Greenwood [27]. The first term on the right side of (3) is the moment about point c generated by gravity. The final term in (3) captures a relatively small centripetal effect that gets ignored when the equations of motion are linearized. The second term captures how lateral acceleration in the ${\hat{\text{e}}}_{2}$ direction affects the lean rate of the pendulum. This is the mechanism by which the steer kinematics affects the lean dynamics.

### Simplified mathematical model: Front steer kinematics

In the simplified bicycle model, the only way that a rider can generate lateral acceleration at point c, term *a*_*c*2_ in Eq 3, is by steering the carriage. Therefore, the next modeling step is to express acceleration *a*_*c*2_ in terms in terms of rider’s steer angle input *δ*, and perhaps time derivatives of the steer angle.

**Fig 6:**
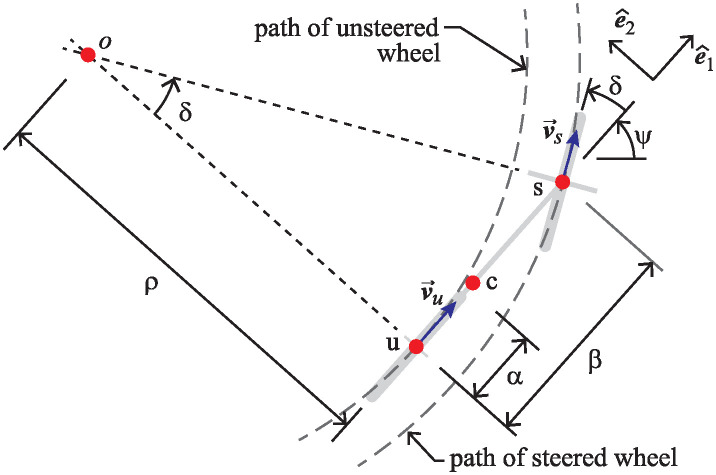
Steer geometry of the front-steered carriage.

To determine the acceleration at point c, we first look at the velocities at points u and s. Referring to Fig 4, one sees that both these points lie on the lean axis, like point c. The point u lies directly below the hub of the unsteered wheel, and directly above the point where the unsteered wheel touches the ground plane. Point s is the location where the vertical steer axis intersects the lean axis. These three points also appear in Fig 6 which show the steer geometry and the curvature of the paths the that points u and s would take, corresponding to steer input, *δ*.

Because the wheels do not slip laterally, the velocity of point u must have magnitude *v* and its direction must align with unit vector ${\hat{\text{e}}}_{1}$:

$$\begin{array}{c} {\vec{v}}_{u}=v\;{\hat{e}}_{1}. \end{array}$$ (4)

Similarly, for the steered wheel, the velocity of point s must have a direction aligned with the “steer line,” oriented at the steer angle *δ* relative to the lean axis:

$$\begin{array}{c} {\vec{v}}_{s}=v_{s}\;\text{cos}\left(\delta \right)\;{\hat{e}}_{1}+v_{s}\;\text{sin}\left(\delta \right)\;{\hat{e}}_{2}. \end{array}$$ (5)

In general, the speed *v*_*s*_ will be different from *v*, and it will not be constant. To determine this relationship, we use the fact that points u and s are a constant distance *β* apart and use concepts of relative velocity and rigid body kinematics [28].

$$\begin{array}{c} {\vec{v}}_{s}={\vec{v}}_{u}+{\vec{v}}_{s/u}=v\;{\hat{e}}_{1}+\beta \dot{\psi }\;{\hat{e}}_{2}. \end{array}$$ (6)

Here, ${\vec{v}}_{s/u}$ denotes the velocity of point s relative to point u, and $\dot{\psi }$ is the yaw rate defined in terms of the heading angle *ψ* illustrated in Fig 6. Reconciling Eqs (5) and (6), one can determine a useful expression for the yaw rate in terms of the steering angle of the carriage.

$$\begin{array}{c} \dot{\psi }=\frac{v\;\text{tan}(\delta )}{\beta }. \end{array}$$ (7)

The yaw rate will be useful in deriving an expression for accelerations of interest.

Since the unsteered wheel moves at a constant rate, the acceleration of point u contains only a lateral centripetal component of the form *v*^2^/*ρ*, where *ρ* is the radius of curvature illustrated in Fig 6 [28]. Some simple trigonometry reveals that *ρ* = *β*/tan(*δ*). Therefore, the acceleration of point u is

$$\begin{array}{c} {\vec{a}}_{u}=\frac{v^{2}}{\beta }\;\text{tan}\left(\delta \right)\;{\hat{e}}_{2}. \end{array}$$ (8)

Now, the term that appears in the balance model (3) is the acceleration of point c, which one can write as

$$\begin{array}{c} {\vec{a}}_{c}={\vec{a}}_{u}+{\vec{a}}_{c/u}={\vec{a}}_{u}+(-\alpha {\dot{\psi }}^{2}\;{\hat{e}}_{1}+\alpha \ddot{\psi }\;{\hat{e}}_{2}), \end{array}$$ (9)

where *α* is the distance between points u and c. Here, again, is the use of relative acceleration: ${\vec{a}}_{c/u}$ is the acceleration of point c relative to point u [28]. Taking the time derivative of Eq (7) gives $\ddot{\psi }=v\dot{\delta }/(\beta \;{\text{cos}}^{2}(\delta ))$. Substituting this, (7), and (8) into (9) produces

$$\begin{array}{c} {\vec{a}}_{cf}=-\frac{\alpha v^{2}}{\beta^{2}}\;{\text{tan}}^{2}\left(\delta \right)\;{\hat{e}}_{1}+(\frac{v^{2}}{\beta }\;\text{tan}\left(\delta \right)+\frac{\alpha v}{\beta \;{\text{cos}}^{2}\left(\delta \right)}\dot{\delta }){\hat{e}}_{2}. \end{array}$$ (10)

The subscript *cf* indicates that this is the acceleration for point c on the front-steered bike.

Note that the first term in the expression above is in the longitudinal ${\hat{\text{e}}}_{1}$ direction and thus has no direct effect on the side-to-side lean dynamics of the pendulum. The second term in Eq (10) is lateral and therefore does impact the lean dynamics.

Note that the lateral ${\hat{\text{e}}}_{2}$ component of ${\vec{a}}_{cf}$ above has two terms. The first, proportional to *v*^2^, is the same centripetal acceleration in Eq (8). The other acceleration term, proportional to *v*, will play an important role in explaining why the rear-steered bicycle is fundamentally different than the front-steered bike.

### Simplified mathematical model: Rear steer kinematics

For the rear-steered bicycle, there is a similar pendulum/carriage model as shown in Fig 5. The direction of travel indicated in Fig 5B confirms that the unsteered wheel is at the front of the bike and the steered wheel is at the back. The direction of the ${\hat{\text{e}}}_{1}$ vector is flipped so that it points in the direction of travel. Also, the direction of the steer angle is flipped so that a positive angle *δ* generates a turn to the left (when facing the direction of travel) just as it did for the front-steered bike. Likewise, the lean angle *θ* for the rear-steered bicycle is defined so that a lean to the left corresponds to a negative value of *θ*, just as it did for the front-steered bicycle. A positive yaw rate still corresponds to a counter-clockwise rotation when viewed from above.

To derive an expression for the acceleration of points on the lean axis is nearly identical to that outlined in the previous subsection for the front-steered bicycle. The results are nearly the same as well. In particular, The acceleration of the (front) unsteered wheel, ${\vec{a}}_{u}$ is the same as in Eq (8).

The acceleration of the (rear) steered wheel is slightly different:

$$\begin{array}{c} {\vec{a}}_{cr}=\frac{\alpha v^{2}}{\beta^{2}}\;{\text{tan}}^{2}\left(\delta \right)\;{\hat{e}}_{1}+(\frac{v^{2}}{\beta }\;\text{tan}\left(\delta \right)-\frac{\alpha v}{\beta \;{\text{cos}}^{2}\left(\delta \right)}\dot{\delta }){\hat{e}}_{2}. \end{array}$$ (11)

The subscript *cr* indicates that the expression is the acceleration of point c on the rear-steered bike. Note that there are two sign differences between this expression and the corresponding acceleration for point c on the front-steered bike in Eq 10. The important sign difference lies in the acceleration term linearly proportional to *v*. As described in an upcoming section, it provides the non-minimum phase behavior that makes the rear steered bike challenging to ride.

### Simplified mathematical model: Linearized equations of motion

The final step in deriving the simplified mathematical model is to combine the pendulum lean dynamics, Eq (3) which depends on lateral acceleration *a*_*c*2_, with the steer kinematic relationships, Eqs (10) or (11) which relate *a*_*c*2_ to the rider’s steer angle input, *δ*, and time derivative, $\dot{\delta }$. In keeping with the stated objective of deriving a linearized model about the upright equilibrium solution $\left(\theta ,\dot{\theta },\dot{\psi },\delta ,\dot{\delta })=(0,0,0,0,0\right)$, one can Taylor expand all nonlinear terms and remove all terms of quadratic order and higher: sin(*θ*) ≈ *θ*, cos(*θ*) ≈ 1, sin(*δ*) ≈ *δ*, tan(*δ*) ≈ *δ*, and ${\dot{\psi }}^{2}\approx 0$.

For the front-steered bike, the result is

$$\begin{array}{c} I_{c1}\ddot{\theta }-mgh\;\theta =mh(\frac{v^{2}}{\beta }\delta +\frac{\alpha v}{\beta }\dot{\delta }). \end{array}$$ (12)

For the rear-steered bike, the model equations become

$$\begin{array}{c} I_{c1}\ddot{\theta }-mgh\;\theta =mh(\frac{v^{2}}{\beta }\delta -\frac{\alpha v}{\beta }\dot{\delta }). \end{array}$$ (13)

The only difference between the two models is the sign of the last term on the right sides of the equations.

It is worth noting that if one treats the pendulum as a single point mass, concentrated at a distance *h* from the lean axis, then (12) is identical to the simplified bike model derived by Timoshenko & Young [29]. It is also equivalent to the first model of Åström et al [2].

## Role of steer kinematics in bicycle stabilization

The simplified bicycle models of Eqs (12) and (13) tell a simple story mathematically. The left sides of the equations are the dynamics of an inverted pendulum. One can think of the right sides of the equations as torques that the rider can apply by turning the handlebar (*δ* and $\dot{\delta }$) in effort to prevent the bicycle from falling over. More specifically, inside the parentheses on the right sides of the equations, one sees linearized versions of the lateral accelerations derived in Eq (10) for the front-steered bike and in Eq (11) for the rear-steered bicycle. It is this steer-induced acceleration that the rider has at their disposal to balance the bike.

The rest of this section presents a simple numerical experiment which shows the lateral acceleration generated by a simple steering maneuver. The qualitative differences in the response to the same input may shed light on why control of the front-steered and rear-steered bikes can be fundamentally different.

### Wheel paths

Suppose that a front-steered carriage (Fig 4) and a rear-steered carriage (Fig 5) are both moving along a nearly straight path at a relatively slow speed of 1.1 m/s. Then suppose that both are given the time-varying steer input defined by the logistic function:

$$\begin{array}{c} \delta \left(t\right)=\frac{A}{1+e^{-kt}}. \end{array}$$ (14)

**Fig 7:**
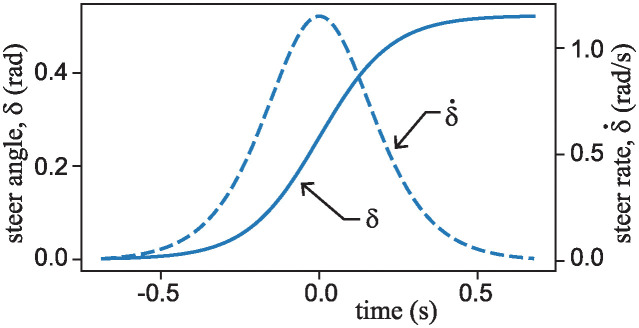
Time dependent steer input *δ* and its time derivative $\dot{\delta }$ used in the numerical experiment. The plot is based on values of *A* = *π*/6 = 30° and $k=8.79\;\frac{1}{s}$.

A plot of *δ* and its first time derivative, $\dot{\delta }$ appear in Fig 7.

Over the time interval shown, the steer angle starts off close to zero, then smoothly but quickly transitions toward another angle *A* = *π*/6 = 30°, corresponding to a nearly steady left turn. The value of *k* in (14) was chosen to be $k=8.79\;\frac{1}{\text{s}}$ so that the 10% to 90% rise time in the steer signal is a half second. If an ordinary, slowly moving bicycle is leaning to the left, a rider might initiate such a steer maneuver in effort to avert a fall.

**Fig 8:**
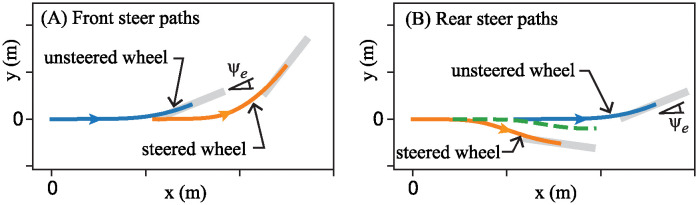
Paths traced out by the two wheels (points u and s) of the carriage as the steer maneuver is executed. (A) Front-steered carriage. (B) Rear-steered carriage. The green dashed curve in (B) shows the path taken by point c, aligned with the center of mass of the pendulum. Plots assumed a speed of *v* = 1.1m/s, and wheel base *β* = 1.09m. The plot of point c in panel B corresponds to a center of mass location *α* = 0.61*β*, the same as Klein’s RSB1. At the final points of the paths shown, two thick gray line segments indicate the locations and orientations of the wheels at that instant.

As the two carriages execute the steer maneuvers prescribed by Eq (14), points u and s of the steered and unsteered wheels trace paths shown in Fig 8. The blue curves trace the paths of the unsteered wheels (point u) on the two carriages, while the orange curves depict the paths of point s corresponding to the steered wheels.

For the case of the front-steered carriage (Fig 8A), the wheels trace out paths that one might expect. At the beginning, when the steer angle is essentially zero, the two wheels track each other along a nearly straight line. But as the handlebar turns the front wheel about the vertical steer axis, the path of the steered wheel immediately begins bending to the left. The unsteered rear wheel follows suit as it gets pulled in the direction of the front wheel.

The corresponding paths of points u and s for the rear-steered carriage, shown in Fig 8B are qualitatively different. As defined in Fig 5 positive steer angle *δ* corresponds to a clockwise rotation of the rear wheel about the vertical steer axis when viewed from above. As a consequence, the rear-steered carriage executes a left turn by first swinging the rear wheel *outward* the right. This action rotates the bike frame and front unsteered wheel counter-clockwise about a vertical axis, setting it up for a leftward turn.

In both cases, the unsteered wheel and bike frame end up changing their heading by essentially the same angle *ψ*_*e*_. But the steered wheels take very different paths to produce this heading change.

### Horizontal acceleration for the front-steered bike

**Fig 9:**
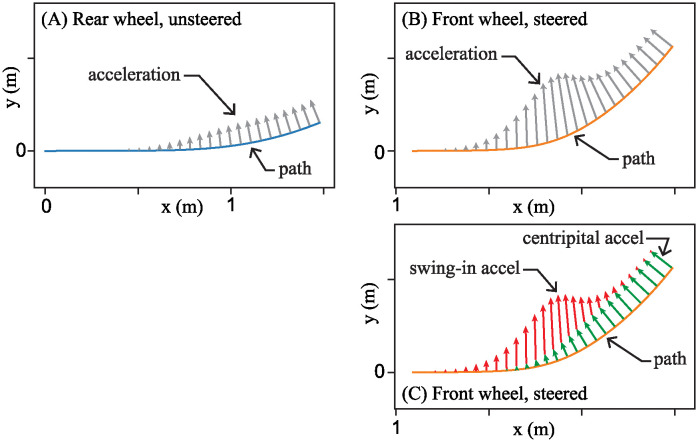
Wheel accelerations of points u and s for the front-steered carriage as it executes the simple steer maneuver. (A) Centripetal acceleration vectors on point u of the rear unsteered wheel. (B) Acceleration vectors on point s of the front wheel are enhanced due to steering action. (C) Two sources of acceleration that enhance each other. Parameters are the same as those listed in Fig 8.

When it comes to balancing the pendulum in the simplified model, Eq (3) tells us that the rider must steer the handlebar in effort to generate lateral acceleration in the direction of the lean. Starting with the front-steered bike, Fig 9 shows the acceleration vectors of points u and s corresponding to the rear unsteered wheel and front steered wheel, respectively, as the rider executes the left turn exhibited in Figs 7 and 8A.

The expression for acceleration of point c on the front-steered carriage, Eq 10, is reproduced here for the reader’s convenience:

$$\begin{array}{c} {\vec{a}}_{cf}=-\frac{\alpha v^{2}}{\beta^{2}}\;{\text{tan}}^{2}\left(\delta \right)\;{\hat{e}}_{1}+(\frac{v^{2}}{\beta }\;\text{tan}\left(\delta \right)+\frac{\alpha v}{\beta \;{\text{cos}}^{2}\left(\delta \right)}\dot{\delta }){\hat{e}}_{2}. \end{array}$$ (15)

It was originally derived as an expression for the point c, a horizontal distance *α* from the rear unsteered wheel. However, one can use it to quantify the acceleration of any point on the lean axis. For, example, setting *α* = 0, one obtains the acceleration at the unsteered wheel, point u. At this location, the first and third term of Eq 15 vanish, leaving just the centripetal component of acceleration, proportional to *v*^2^/*β*. All the acceleration vectors in Fig 9A are perpendicular to the path of the rear wheel and point toward the center of curvature. As the steer angle *δ* increases in Fig 7, the radius of curvature of the rear wheel’s path gets smaller, and the magnitude of the centripetal acceleration increases.

One can obtain the expression for acceleration of point s corresponding to front steered wheel by setting *α* = *β* in Eq 15. In this case, all three terms participate. Fig 9B shows acceleration vectors for the steered wheel as it traces its path. Like the acceleration vectors for the unsteered wheel, they point inward, toward the center of the turn.

In the last panel, Fig 9C shows the acceleration of the front wheel split into two parts. One part is the centripetal acceleration, depicted by green arrows in the figure, and expressed by the two terms proportional to *v*^2^*δ*/*β* in Eq 15. This part of the acceleration is due to the velocity of point s changing its direction as it moves along the path. The other part of acceleration, proportional to $v\dot{\delta }$, is depicted by red arrows in Fig 9C. In the current study, this component is called “swing-in” acceleration. It appears in the acceleration because turning the front wheel of a front steer bike directs more of the wheel’s motion *v* into the ${\hat{\text{e}}}_{2}$ direction. By placing the vectors tip-to-tail, it is easy to see how the vectors reinforce each other to enhance the acceleration at point s.

At point c, coinciding with the center of mass of the pendulum, parameter *α* is somewhere between 0 and *β*. The two parts of the lateral acceleration still reinforce each other, though the swing-in portion is not as strong. To generate lateral acceleration that balances a front-steered bike appears to be a straightforward task. The rider should simply turn into the direction that they wish to accelerate.

### Horizontal acceleration for the rear-steered bike

For the rear-steered bicycle, the reader may recall, there are sign changes in the acceleration. Eq (11) is reproduced here for the reader’s convenience.

$$\begin{array}{c} {\vec{a}}_{cr}=\frac{\alpha v^{2}}{\beta^{2}}\;{\text{tan}}^{2}\left(\delta \right)\;{\hat{e}}_{1}+(\frac{v^{2}}{\beta }\;\text{tan}\left(\delta \right)-\frac{\alpha v}{\beta \;{\text{cos}}^{2}\left(\delta \right)}\dot{\delta }){\hat{e}}_{2}. \end{array}$$ (16)

**Fig 10:**
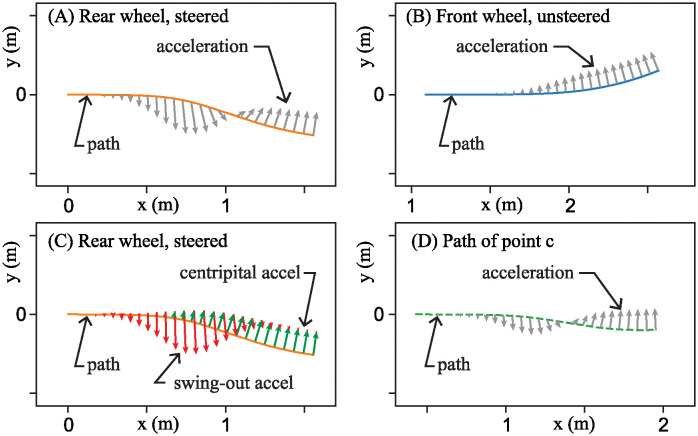
Wheel accelerations of points u and s for the rear-steered carriage as it executes the simple steer maneuver. (A) As the carriage executes a left turn, acceleration vectors of point s of the rear-steered wheel. (B) Acceleration vectors of point u of the front unsteered wheel. (C) Two sources of acceleration that oppose each other on the rear-steered wheel. (D) Acceleration vectors on point c aligned with pendulum center of mass. Parameters are the same as those listed in Fig 8.

For *α* = 0, the expression reduces to the single term representing centripetal acceleration of the unsteered front wheel. The corresponding acceleration vectors are shown in Fig 10B. The vectors point inward, perpendicular to the path; the magnitude gets larger as the steer angle increases and the radius of curvature gets smaller. We encountered a similar behavior in the unsteered wheel of the front-steered bike (Fig 9A). It is what unsteered wheels do.

One look at Fig 10A, however, reveals that the steered wheel on the rear-steered bike is qualitatively different from the steered wheel of a front-steered bike. During the simple steering maneuver in which the handlebar is smoothly turned to the left, the steered wheel starts accelerating to the right and then switches direction and ends up accelerating to the left.

This happens because, in order for the rear-steered bike to execute a left turn, the rear wheel must first swing outward so that the whole frame, including the rider, can turn to the left. All three terms of Eq (16) participate in this observance. As was done previously, Fig 10C shows the acceleration split into two parts. The green arrows are the centripetal part, proportional to *v*^2^/*β* due to the fact that the rear wheel eventually does turn to the left. The other part of the acceleration, proportional to $v\dot{\delta }$, is due to the rear wheel’s change in velocity toward the right as it swings outward during the initial part of the turn. This is labeled “swing-out acceleration” in the figure.

Fig 10C illustrates how the swing-out acceleration opposes the centripetal acceleration. It is these two opposing components of the acceleration, in contrast to the reinforcing accelerations for the front-steered bike, that can make it exceedingly difficult to balance a rear-steered bicycle.

What matters most, in term of balancing the inverted pendulum/bike, again, is the acceleration at point c. For Klein’s RSB1, the value of *α* is about 0.61*β*. Fig 10D shows the acceleration of point c for this value of *α* during the same turn maneuver. Again, it shows a significant cancellation effect between the swing-out and centripetal component parts acceleration.

### Consequences for control

The standard approach for balancing a bicycle (that is not self-stable) is for the rider to detect the direction in which the bike is leaning and then to steer the handlebar into the direction of the lean. For the front-steered bike, Fig 9 illustrates, to a large degree, why that approach is successful. For example, if the bicycle is leaning to the left, then a leftward turn of the handlebar will generate two types of acceleration to the left, both reinforcing each other. If an appropriate amount of such steer input is provided, it will arrest the lean and upright the bike.

For the rear-steered bike leaning to the left, such a steer input might have a dire consequence. Turning the handlebar to the left would initially produce a swing-out acceleration to the right which would hasten the fall. Perhaps the centripetal acceleration could kick in soon enough for the rider to avert a crash. Maybe not.

The input response behavior exhibited in Fig 10A, 10C and 10D in which the opposite-sign swing-out acceleration appears first before the correct-sign centripetal acceleration takes over is the signature feature of what control engineers call non-minimum phase systems, or inverse response [30]. In general, such non-minimum phase systems might not be too bothersome [31]. However, if the timescale at which the non-minimum phase behavior resolves itself is roughly equal to the timescale of an unstable mode of the open-loop system, then there are inherent difficulties in controlling the system. This is exactly how Åström et al [2] and Åström [12] describe the difficulty in controlling Klein’s RSB1, albeit in the language of right half-plane open-loop zeros (*z*, non-minimum phase zero timescale) and open-loop poles (*p*, unstable mode timescales), instead. They state that if such an open-loop zero and open-loop pole coincide, then the system loses controllability (see Ogata [32]). Furthermore, Åström [12] argues that a system lacks sufficient robustness properties as long as the zero-to-pole ratio lies in the range 0.25 < *z*/*p* < 4.

As one starts thinking about strategies for balancing the “unridable” rear-steered bicycle, one of many things to think about is the role of speed. Recall that the swing-out acceleration is proportional to speed *v*, while the centripetal acceleration is proportional to *v*^2^. Therefore, the proportion of the two types of opposing accelerations changes as the speed of the bike changes.

### Testing modeling assumptions

The previous section provided an explanation, based on counteracting effects of centripetal acceleration and “swing-out” acceleration, for why Klein’s Rear-Steered Bike I (RSB1) might be unridable. However, the argument is based on a simplified model which ignored certain characteristics of real bicycles such as: (A1) gyroscopic effects of rotating wheels; (A2) caster effects on the steered wheel along with a tilted steer axis; (A3) the effect of the handlebar/fork/wheel assembly having a center of mass offset from the steer axis; and (A4) details of the way in which mass is distributed throughout the bike.

In the section titled Supporting Information, there is a link to the S1 Appendix which provides a detailed comparison of the simplified model of the rear-steered bike (Eq (13)) to the validation model in Eq 2. There are two main conclusions.

1. **The simplified model of the rear-steered bike is equivalent to the validation model (2) subject to Assumptions A1 through A4.** The structure of the equations are the same, and all the remaining terms are identical. This is important because the starting point for deriving the bike models for this article is a pendulum attached to a carriage with training wheels, as depicted in Figs 4 and 5. In contrast, the starting point for the validation model derived by Meijaard [20] is the Whipple-Carvallo bicycle which better resembles a typical bicycle. In this article, we formulated the bike balancing problem in terms of the pendulum/carriage because it better illustrates that the role of steering (the role of the carriage) is to provide a lateral acceleration to the pendulum. The result shows that the pendulum/carriage is mechanically equivalent to a Carvallo-Whipple-type bicycle for which the assumptions are valid.
2. **The assumptions A1 through A4 represent quantitatively small effects that do not qualitatively alter the counteracting actions of rear-wheel steering highlighted previously.** Upon restoring the gyroscopic effect, caster effects, a tilted steer axis, and allowing for more general mass distribution representative of Klein’s RSB1, the S1 Appendix describes a numerical experiment similar to that carried out in Fig 10. When given the steer input shown in Fig 7, the full validation model for the rear-steered bike exhibits the same non-minimum phase behavior as the simplified rear-steered bike model. The cross-over time at which the steer input switches from pushing in the wrong direction to pulling in the correct direction is about the same as the Simplified Model. Gyroscopic effects do appear in the two terms proportional to *v*^2^*δ* and to $v\dot{\delta }$. However, the centripetal and swing-out accelerations are the dominant effects on the right side of the pendulum equation.

Upcoming sections on controllability analysis and devising riding strategies will continue to focus on the Simplified Model since it still offers the clearest way to think about the dynamics of the rear-steered bike.

## State space analysis

To gain insight into how an aspiring rider might exploit the competing accelerations in effort to stabilize the rear-steered bike, we turn to a state space control perspective. In doing so, we take the linearized mathematical models derived previously (12) and (13), and re-write them in standard state space form:

$$\begin{array}{c} \dot{x}\left(t\right)=A\;x\left(t\right)+B\;u\left(t\right), \end{array}$$ (17)

where $\omega =\dot{\theta }$,

$$\begin{array}{c} x\left(t\right)=[\begin{array}{c} \theta (t)\\ \omega (t)\\ \delta (t) \end{array}],\;\text{and}\;A=[\begin{array}{ccc} 0 & 1 & 0\\ \frac{mgh}{I_{c1}} & 0 & \frac{mhv^{2}}{\beta I_{c1}}\\ 0 & 0 & 0 \end{array}]. \end{array}$$

The 3 × 1 ***B*** matrices for the for the front-steered and rear-steered bicycle models become

$$\begin{array}{c} B=B_{f}=[\begin{array}{c} 0\\ \frac{\alpha mhv}{\beta I_{c1}}\\ 1 \end{array}],\;\text{and}\;B=B_{r}=[\begin{array}{c} 0\\ -\frac{\alpha mhv}{\beta I_{c1}}\\ 1 \end{array}], \end{array}$$ (18)

respectively. Note that the control input *u* in (17) represents the steer rate, $\dot{\delta }$. The steer angle *δ*, itself, is included in ***x*** and thus is treated as a state.

For the lean dynamics of our simplified bike models, the state space is three dimensional. Within this three dimensional space, one can think of the right side of (17) as a vector field. Solutions to the differential Eq (17) are integral curves in state space that are everywhere tangent to the vector field.

### Drift dynamics

When we set *u*(*t*)≡0, we are left with only the natural dynamics of the linearized bike model with the handlebar held at a constant steer angle:

$$\begin{array}{c} {}[\begin{array}{c} \dot{\theta }\left(t\right)\\ \dot{\omega }\left(t\right)\\ \dot{\delta }\left(t\right) \end{array}]=[\begin{array}{ccc} 0 & 1 & 0\\ \frac{mgh}{I_{c1}} & 0 & \frac{mhv^{2}}{\beta I_{c1}}\\ 0 & 0 & 0 \end{array}][\begin{array}{c} \theta (t)\\ \omega (t)\\ \delta (t) \end{array}]. \end{array}$$ (19)

One may call this the drift dynamics, and it is the same for both the front-steered and rear-steered bikes.

**Fig 11:**
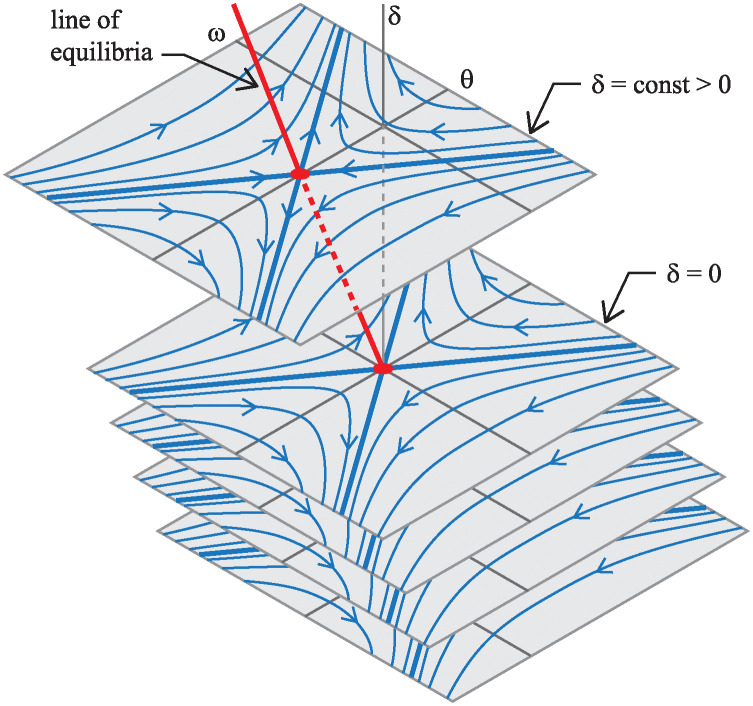
Foliation of phase portraits for the bicycle model’s drift dynamics (19).

Since the bottom equation in (19), states that $\dot{\delta }=0$, the state space is foliated by invariant planes: *δ* = const, when *u* ≡ 0 as shown in Fig 11. Each leaf of the foliation corresponds to the bike being held at a different, constant steer angle *δ*. Furthermore, Eq (19) possesses an entire line of equilibria: (*θ*, *ω*) = (−*δv*^2^/*βg*, 0). Thus, for each constant steer angle *δ*, there is a corresponding equilibrium lean angle *θ* for which the gravitational moment exactly balances an effective moment due to a constant centripetal acceleration.

Aside from the zero eigenvalue corresponding to the line of equilibria, the 3×3 matrix ***A*** in (19) has two other real eigenvalues: $\lambda =\pm \sqrt{mgh/I_{c1}}$, one positive and one negative. Therefore, each equilibrium of the drift dynamics is of saddle type, the typical linearized dynamics about an equilibrium of an inverted pendulum. The positive real eigenvalue accounts for the instability corresponding to bike falling to either side. In each *δ* = const invariant plane of the drift dynamics, the stable and unstable eigenspaces are in directions tangent to eigenvectors $v_{+}={\left[1\;\;\sqrt{mgh/I_{c1}}\;\;0\right]}^{T}$ and $v_{-}={\left[-1\;\;\sqrt{mgh/I_{c1}}\;\;0\right]}^{T}$, respectively.

Fig 11 shows phase portraits for the saddle equilibria on several different *δ* = const slices. Notice, from the lack of speed dependence in the expressions above, the phase portrait on each leaf of the foliation looks exactly the same at different speeds. It is the line of equilibria that gets more skewed as speed increases.

### Adding the control vector field

To more easily visualize the effect of control, one can define a new variable $\tilde{\theta }$ as

$$\begin{array}{c} \tilde{\theta }\left(t\right)=\theta \left(t\right)+\frac{v^{2}}{g\beta }\delta . \end{array}$$ (20)

This transformation shifts the line of equilibria to the *δ* axis, where $\left(\tilde{\theta },\omega )=(0,0\right)$. As a result, the linearized model with control input, Eq (17), can be re-written as

$$\begin{array}{c} \dot{\tilde{x}}\left(t\right)=\tilde{A}\;\tilde{x}\left(t\right)+\tilde{B}\;u\left(t\right), \end{array}$$ (21)

where

$$\begin{array}{c} \tilde{x}\left(t\right)=[\begin{array}{c} \tilde{\theta }\left(t\right)\\ \omega (t)\\ \delta (t) \end{array}],\;\;A=[\begin{array}{ccc} 0 & 1 & 0\\ \frac{mgh}{I_{c1}} & 0 & 0\\ 0 & 0 & 0 \end{array}],\;\;{\tilde{B}}_{f}=[\begin{array}{c} \frac{v^{2}}{g\beta }\\ +\frac{\alpha mhv}{\beta I_{c1}}\\ 1 \end{array}],\;\;{\tilde{B}}_{r}=[\begin{array}{c} \frac{v^{2}}{g\beta }\\ -\frac{\alpha mhv}{\beta I_{c1}}\\ 1 \end{array}]. \end{array}$$ (22)

**Fig 12:**
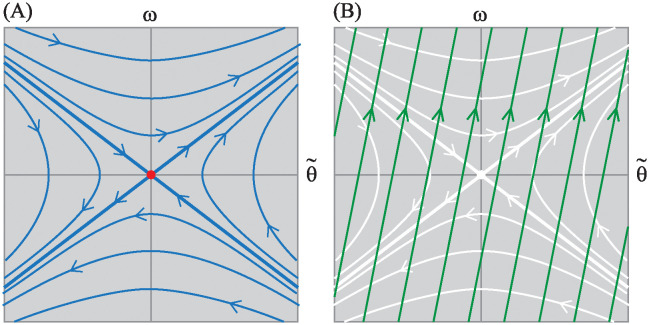
Integral curves. (A) For the drift vector field $\tilde{\text{A}}\tilde{\text{x}}$. (B) For the control vector field $\tilde{\text{B}}u$ when *u* is a positive constant.

Here, ${\tilde{\text{B}}}_{f}\;u(t)$ and ${\tilde{\text{B}}}_{r}\;u(t)$ are the control matrices for the front-steer and rear-steer bike models respectively. Notice that performing this speed-dependent transformation in (20) removed the *v* dependence in the drift vector field $\tilde{\text{A}}\tilde{\text{x}}$. Furthermore, because the new coordinate system is aligned with the line of equilibria, the phase portraits on each leaf of the foliation stack up, as carbon copies, right on top of each other. Because the right column of $\tilde{\text{A}}$ consists of all zeros, there is no steer angle dependence in the new expression of the drift dynamics. One can investigate stability, controllability, and stabilizability by considering only the two dimensional $\tilde{\theta },\omega$ subspace, where the integral curves of the drift dynamics (*u*(*t*) ≡ 0) look like those shown in Fig 12A.

Furthermore, Fig 12B shows integral curves of $\dot{\tilde{\text{x}}}=\tilde{\text{B}}\;u$, for the control vector field projected onto the *δ* = const, $\left(\tilde{\theta },\omega \right)$ subspace. The linearized bicycle model never actually produces solutions like those shown in Fig 12B. However, the curves are useful in that they depict the direction in which the rider can “push” the $\left(\tilde{\theta },\omega \right)$ state of the bike by producing a $\dot{\delta }$ steer input. In this case, the green arrows indicate the direction a rider can “push” the state with a positive $\dot{\delta }$. A negative $\dot{\delta }$ would push in the opposite direction in state space. By overlaying integral curves of the control vector field on top of integral curves of the drift vector field as in Fig 12B, one can see, graphically, what type of steer input would push the state toward the stable eigenspace of the natural drift dynamics.

In the formulation of (21), it is worth noting that all the speed dependence *v*, appears in the control vector field $\tilde{\text{B}}\;u(t)$. The top element of the $\tilde{\text{B}}$ matrix is proportional to *v*^2^, corresponding to the centripetal acceleration produced by steer kinematics. The middle element of $\tilde{\text{B}}$ is linearly proportional to *v*, corresponding to the “swing-in” (front-steered bike) or “swing-out” (rear-steered bike) part of the lateral acceleration. And since the two terms depend differently on *v*, the direction of the control vector field changes for different bike speeds.

### Controllability and stabilizability

Upon constructing the standard controllability matrix $\tilde{\text{R}}=[\tilde{\text{B}}\;\tilde{\text{A}}\tilde{\text{B}}\;{\tilde{\text{A}}}^{2}\tilde{\text{B}}]$, one finds that it becomes singular for bicycle speed *v* = 0 and for $v=\alpha \sqrt{mgh/I_{c1}}$. The result holds for both front-steered and rear-steered bicycles.

It makes sense that controllability is lost for our bike model when *v* = 0. When the Simplified Model of the bike is not moving, it cannot generate lateral acceleration. More puzzling is that there is a forward speed, well within the range of what would be considered normal cycling speeds, for which the front-steered bike loses controllability.

#### Front-steered bicycle model

Controllability [32] refers to the property of being able to steer *any* initial state to *any* final state using some combination of the drift vector field and a time-varying control vector field. The specific control vector field and the drift vector shown in Fig 12 would have a full-rank $\tilde{\text{R}}$, corresponding to a controllable system.

**Fig 13:**
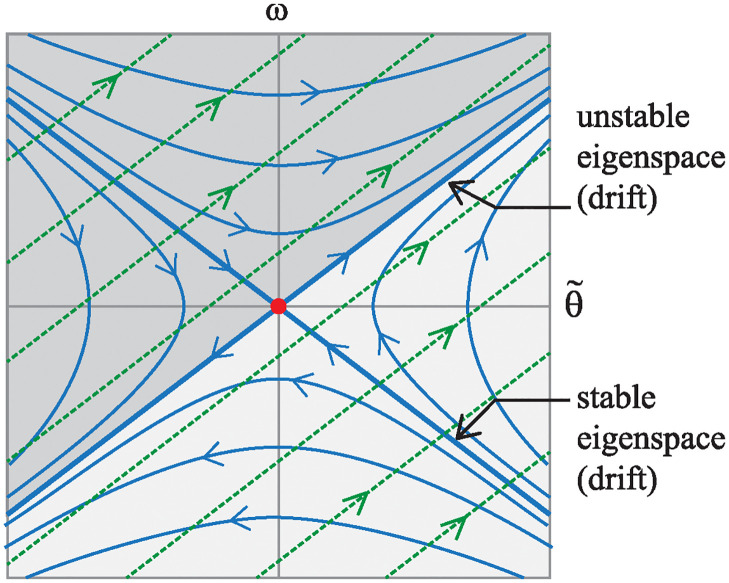
Loss of controllability, from a state space perspective, for the front-steered bike model. Integral curves projected onto a *δ* = const plane for the drift vector field and for the control vector field for the front-steered bike at the speed where the controllability is lost. Integral curves for the drift vector field are solid and shown in blue. Integral curves for the control vector field are dashed and shown in green.

In contrast, consider the circumstance shown in Fig 13 depicting what happens to the state space integral curves when the front-steered bike travels at speed $v=\alpha \sqrt{mgh/I_{c1}}$. Here we see that ${\tilde{\text{B}}}_{f}$ aligns with the unstable eigenspace of the drift vector field.

The unstable eigenspace of the drift dynamics shown in the figure is a projection onto a single *δ* = const plane. In the full three dimensional state space of Eq 21, the unstable eigenspace is a two dimensional, invariant surface that separates the state space into two parts that are indicated by two different shades of gray in Fig 13. In the absence of control, $u=\dot{\delta }=0$, solutions in the dark gray side, never “drift” to the light gray side and vice versa. And since the control vector field acts tangent to the unstable eigenspace, control action is unable to “push” the dynamic state transverse to the surface. It is impossible to use control to steer states on one side of the boundary to the other. Controllability is lost.

To the bicycle rider interested maintaining balance, though, this result is inconsequential. Fig 13 shows that the control vector field does act transverse to the stable eigenspace. To keep the bicycle from falling to one side, the rider can apply a steer input that pushes the state toward the stable eigenspace, then allow the stable component of the drift dynamics to carry the state toward the line of equilibria at $\left(\tilde{\theta },\omega )=(0,0\right)$. Although the linearized model of the front-steered bike loses controllability at one specific speed, it is still *stabilizable* [32] at all forward speeds greater than zero. The result aligns with one’s every-day experiences of riding a front-steered bicycle.

#### Rear-steered bicycle model

The rear-steered bikes also loses controllability at the speed:

$$\begin{array}{c} v=v_{cr}=\alpha \sqrt{mgh/I_{c1}}. \end{array}$$ (23)

**Fig 14:**
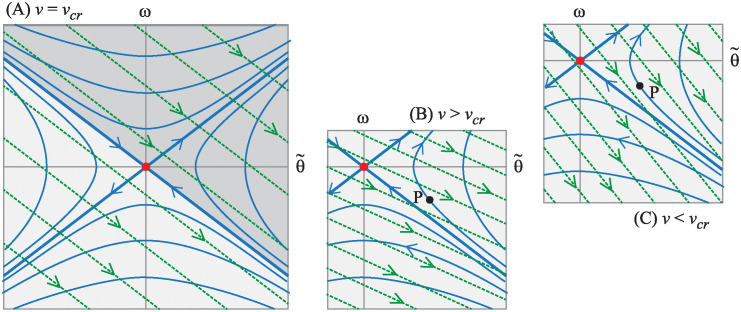
Loss of controllability and crossover for the rear-steered bike model. (A) Integral curves projected onto a *δ* = const plane for the drift vector field and for the control vector field for the rear-steered bike at the speed where the controllability is lost. Integral curves for the drift vector field are solid and shown in blue. Integral curves for the control vector field are dashed and shown in green. (B) Same curves, but for a speed slightly faster than the crossover speed. (C) Same curves, but for a speed slightly slower than the crossover speed.

However, the consequences for the rear-steered bicycle are more dire. At this speed that we call the crossover speed (*v*_*cr*_), the control vector field for the rear-steered bike lines up with the stable eigenspace as shown in Fig 14A. Therefore, like the loss of controllability for the front-steered bike model, one can use the stable eigenspace to separate state space into a darkly shaded half and a lightly shaded half. At linear order, the control is unable to push states from one side of the state space to the other. Controllability is lost.

However, since ${\tilde{\text{B}}}_{r}$ is tangent to the stable eigenspace, it becomes impossible to use control to arrest the unstable dynamics. One cannot steer the state toward the stable eigenspace. As a consequence, one cannot use steer input to keep the bike balanced. Therefore, at the crossover speed *v* = *v*_*cr*_, the rear-steered bike lacks stabilizability, in addition lacking controllability. Connecting this result to the physics of the problem, one can say that loss of stabilizability occurs for the rear-steered bike when the centripetal acceleration (proportional to *v*^2^) and the swing-out acceleration (proportional to *v*) counteract in a way that makes it impossible to actively balance a bike using steer input.

Fig 14B shows overlays of the integral curves of the control vector field on top of integral curves of the drift dynamics. The bike speed for this case is slightly faster than the crossover speed. To describe what is happening here, suppose that at some time, the state of the bicycle is at the point labeled P in the figure. Since a positive control input $\dot{\delta }$ creates a “push” in the direction of the green arrows (on the dashed curves) in state space, the rider at state P should choose a sufficiently large *negative* steer rate ($\dot{\delta }<0$) to drive the system’s dynamics back toward the stable eigenspace. Once close to the stable eigenspace, the natural drift dynamics of the bike will pull the state toward the equilibrium.

If one thinks about what this means physically, point P corresponds to the rear-steered bike leaning too far to the right, given the current lean rate *ω* and steering angle *δ*. Furthermore, if the rider does not respond to this situation, the bike and rider will fall to the right. As just discussed regarding the portrait in Fig 14B, the appropriate action for the rider to take is one that generates a negative steer rate $\dot{\delta }$. For $\dot{\delta }<0$, the rear wheel turns to the left, causing the entire bike to turn to the right. This is the normal response for attempting to stabilize a leaning bike: turn the bicycle into the direction of the lean.

In contrast, look at the overlaid integral curves in Fig 14C where the bike is traveling slightly slower than the crossover speed. Again, one can consider the same state P corresponding to a bike/rider leaning too far to the right. Arrows of the integral curves of the control vector field in this case indicate that the rider should immediately respond with a sufficiently large *positive* steer rate, $\dot{\delta }>0$. This is the exact opposite action the rider should take when the bike is traveling slightly faster than *v*_*cr*_.

At slower speeds, the swing-out acceleration has a stronger effect than its centripetal counterpart. Physically, the $\dot{\delta }>0$ response corresponds to the rider executing a left turn, away from the lean of the bike, but doing so in a way that causes the rear-steered wheel to initially swing toward the right to arrest the fall of the right-leaning pendulum.

One final observation to make regarding Fig 14B and 14C is that when the bike speed is close to the crossover speed, the control vector field is mostly aligned with the stable eigenspace. Because of this, a relatively large amount of steer input is required to counteract the unstable dynamics.

For the front-steered bike, there are no such complications. The control vector field ${\tilde{\text{B}}}_{f}$ is well aligned with the unstable drift dynamics that one is trying to thwart. The steering strategy for the front-steer bike is essentially the same, qualitatively, regardless of speed.

Finally, it is worth mentioning that loss of controllability in the state space representation promoted here is equivalent to overlapping open-loop poles and zeros in a transfer function representation of the dynamics. (See Ogata [32].) For the front-steered bike, the open-loop zero is in the left half-plane. So, it cancels a stable open-loop pole when controllability is lost. In contrast, for the rear-steered bike, the open-loop zero is in the right half-plane. The cancellation (or near-cancellation) with an unstable pole, *z*/*p* ≈ 1, leads to the robustness problems that Åström [12] warns of.

## Strategies for riding the rear-steered bicycle

It is clear that the ability to stabilize and balance the rear-steered bike model is dependent on the component of vector ${\tilde{\text{B}}}_{r}$ that is aligned with the unstable eigenspace of the uncontrolled drift dynamics. With this in mind, one can decompose ${\tilde{\text{B}}}_{r}$ into eigen-components:

$$\begin{array}{c} {\tilde{B}}_{r}=b_{u}\;v_{+}+b_{s}\;v_{-}+b_{\delta }\;v_{0}. \end{array}$$

Here, **v**_+_, **v**_−_, and **v**_0_ are eigenvectors of $\tilde{\text{A}}$ corresponding to the positive, negative, and zero eigenvalues. In this case, *b*_*u*_ is given by

$$\begin{array}{c} b_{u}=\frac{1}{2}(\frac{v^{2}}{g\beta }-\frac{\alpha v}{\beta }\sqrt{\frac{mh}{gI_{c1}}}). \end{array}$$ (24)

**Fig 15:**
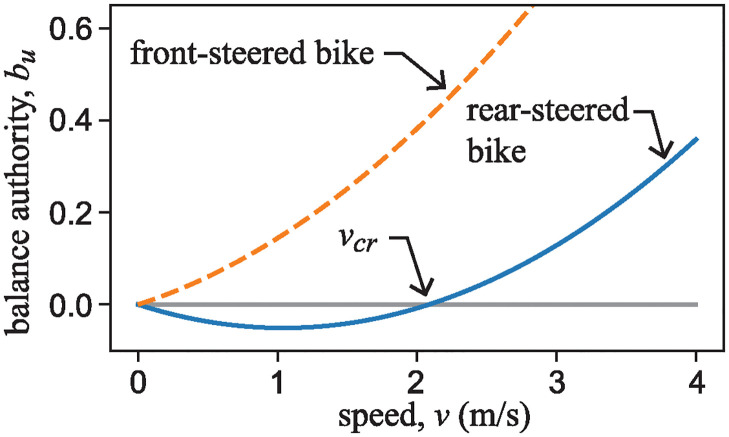
Plot of balance authority, *b*_*u*_ as a function of bike speed, *v*. The solid curve is *b*_*u*_ given by (24) for the rear-steered bike, while dashed curve is the corresponding measure for a typical front steer bike. Parameters *β* = 1.09m and *α*/*β* = 0.61 were measured from Klein’s RSB1, while parameters *m* = 94kg, *h* = 0.861m, *g* = 9.81N/kg, *I*_*c*1_ = 80.8kg m^2^ were obtained from [20]. *b*_*u*_ for the front steer bike is determined by changing the sign of the second term in (24), and using a value of *α*/*β* = 0.34.

The quantity *b*_*u*_ is dimensionless and provides a measure of how much authority the rider has, through steering input, to act against the unstable inverted pendulum dynamics of the bike. The solid curve in Fig 15 is a plot of this “balance authority” as a function of bike speed, *v*. To generate the plot, numerical values for parameters *α* and *β* were measured while the author was sitting on Klein’s RSB1. Other parameters *h*, *m*, and *I*_*c*1_ were based on the benchmark bicycle of Meijaard et al. [20].

The plot shows that for *v* = 0, the balance authority is zero. This is due to the control vector $\tilde{\text{B}}$ being identically zero when the bike has zero speed. When not moving, it is impossible for the simplified bike model to generate a lateral acceleration, using steer input, that would have an effect on a leaning pendulum.

Since the control vector, $\tilde{\text{B}}$, has parts that are linear and quadratic in speed *v*, depending on whether they derive from swing-out or centripetal acceleration effects, the expression for *b*_*u*_ in (24) has the same linear plus quadratic structure. As the speed *v* increases from zero, the value of *b*_*u*_ first becomes negative and then crosses over to positive values at the crossover speed, *v*_*cr*_. The zero value of *b*_*u*_ at the crossover speed corresponds to the loss of stabilizability. As one varies the *v* parameter through the crossover speed, the input/output behavior of the system switches from one for which the swing-out acceleration is more important to another for which centripetal acceleration plays the most important role. Crossing the crossover threshold requires a change in strategy for balancing the bike.

For comparison, the analogous balance authority *b*_*u*_ for the front steer bike is also shown in Fig 15 as a dashed curve. The curve quickly departs from zero as the speed *v* increases. Here, the balance authority is always positive because the “swing-in” and centripetal components of acceleration reinforce each other for the front-steered bike at all forward-moving speeds.

### Strategy 1: Ride fast

In [13], Åström suggests a ride-fast strategy for balancing the rear-steered bike. By rapidly accelerating the bike (past the crossover speed, *v*_*cr*_), Åström argues that one can quickly create separation between the non-minimum phase zero and open-loop unstable pole, allowing for robust stabilization. From the perspective advanced in this article, such an approach would quickly propel the rider into a regime in which the centripetal part of lateral acceleration, proportional to *v*^2^, dominates the swing-out portion, proportional to *v*. A rider in such a situation would have ample balance authority (Fig 15), and would be able to adopt a balancing strategy of simply turning into the direction of lean, just like balancing a traditional front-steered bicycle.

**Fig 16:**
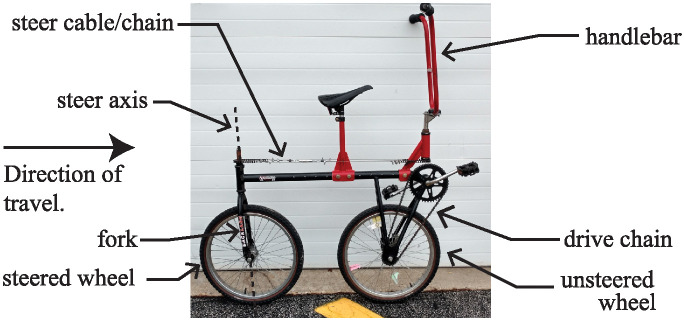
The “Easy Rear-Steered Bicycle” (EZRSB). Built by the author’s students. The wheel base is *β* = 0.62m; and center of mass location *α* = 0.2*β*, leading to a crossover speed of approximately *v*_*cr*_ = 0.4m/s.

**Fig 17:**
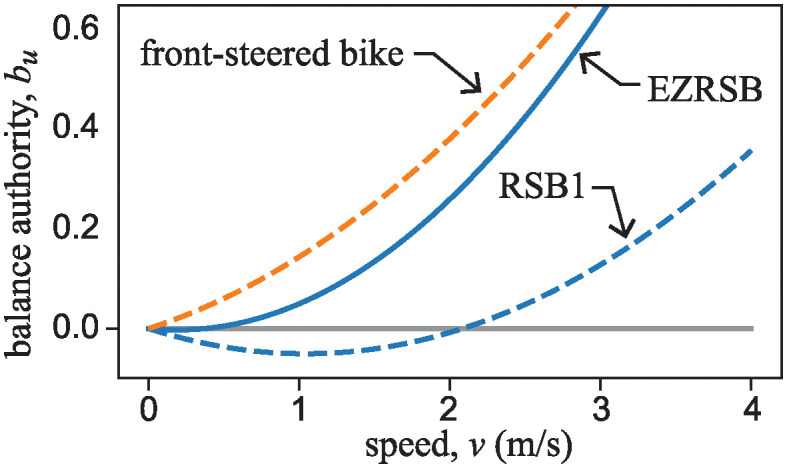
Balance authority for the “Easy Rear-Steered Bicycle” (EZRSB). Comparisons are provided to the same measure on a typical front-steered bicycle (orange dashed), and to Klein’s “unridable” RSB1 (blue dashed).

This strategy actually works for the rear-steered bicycle shown in Fig 16, nicknamed EZRSB (easy rear-steered bike), which is patterned off a similar rear-steered bike that Klein built, which he called “Rear-Steered Bike II” [1, 2]. Notice from the photo that the seat of the EZRSB bike is relatively far forward, moving the center of mass closer to the front unsteered wheel. This significantly reduces the parameter *α*. Also, the bike’s saddle is relatively high, increasing the rotational inertia, *I*_*c*1_, about the lean axis. Combined, these two effects tend to lower the crossover speed to approximately 0.4 m/s. This is about 80% lower than that of Klein’s “unridable” RSB1, making the crossover threshold much easier to surpass. The corresponding measure of balance authority, *b*_*u*_, as a function of speed for the EZRSB is provided in Fig 17, with comparisons to a typical front-steered bike and the RSB1.

In the author’s experience of riding and observing students ride the EZRSB, if one does not get a strong initial push, it is difficult to pedal fast enough before falling. This is shown in video S2 Video. Compared to the front-steered bike, the balance authority in Fig 17 for the EZRSB is very close to zero over a range of relatively slow speeds. However, if that initial push is sufficient to propel the bike and rider past a certain speed threshold, balancing the Easy Rear-Steered Bike is almost as simple as riding a traditional front-steered bicycle. One cannot ride EZRSB without hands on the handlebar; it is not self-stable. But since lateral acceleration of the bike at these higher speeds is dominated by centripetal effects, riding the EZRSB requires no additional rider training, provided that the steer chain is in the figure eight configuration as shown in Fig 2. In the author’s presence, every rider who has had the fortitude to give the bike a strong enough initial push has been able to succeed at riding it in a controlled manner.

A video of the author riding EZRSB is provided in S3 Video. Although the bicycle itself looks odd, the way that the rider initiates leans, executes turns, and returns the bike to a non-leaned state seem natural. By observing the rotation rate of the wheels, one can estimate the speed of the bike at the beginning of the video at about 2.1 m/s. This is a factor of 5.25 larger than the estimated crossover speed of *v*_*cr*_ = 0.4 m/s, yielding an open-loop zero to pole ratio of *z*/*p* = 5.25. This is well past the ratio *z*/*p* = 4 that Åström [12] recommends for robust control. At this speed, the dimensionless balance authority measure was estimated to be about *b*_*u*_ = 0.41.

During experimentation, the author was able to ride the EZRSB as slow as 1.1 m/s, about 2.75 times larger than the estimated crossover speed. At a speed of 1.1 m/s, the estimate of the balance authority, according to Eq (24), is approximately *b*_*u*_ = 0.06. To be clear, this experimental measure of the author’s ability to balance the EZRSB by first traveling at a high enough speed where it was rather easy to balance the bike, and then slowing down until maintaining balance was no longer possible. S4 Video shows EZRSB operating near this threshold. Accompanying the loss of ability to keep the bike balanced, one also loses directional control.

Another example of a rear-steered bicycle for which the “Ride Fast” strategy works well is a BMX bike trick called the “Fakie”, in which riders travel backwards. The interested reader can search on YouTube and find instructional videos that urge riders to keep their weight over the unsteered wheel. This makes sense, according to the stabilizability analysis presented here. Keeping one’s weight over the unsteered wheel keeps *α* small and thus reduces the crossover speed.

What has proven to be a good strategy for the EZRSB with its center of mass close to the unsteered wheel (*α* small), is not a viable approach for Klein’s “unridable” Rear-Steered Bike I (RSB1). Because the center of mass on RSB1 is closer to steered wheel (*α* large), there is a larger “swing out” component of the lateral acceleration. As a consequence, the estimated crossover speed for RSB1 is approximately 2.1 m/s, compared to 0.4 m/s for EZRSB. If achieving a balance authority of *b*_*u*_ = 0.06 is imperative for RSB1, as it was for EZRSB, then RSB1 would need an initial push of 2.6 m/s to get it over the threshold where a strategy based on centripetal acceleration would work. If having a zero-to-pole ratio *z*/*p* = 2.75 is important, then the initial push on RSB1 would have to produce a speed of 5.8 m/s in less than the fraction of a second that it takes to fall. This is two to five times larger than the initial impulse needed to get the EZRSB going. If one’s goal is to satisfy the requirements of Klein’s Rear-Steered Bicycle Challenge, then this does not appear to be a viable strategy. The author and his students have not been able to generate a large enough initial speed on RSB1 for which the “Ride Fast” strategy could work.

### Strategy 2: Ride slowly and exploit the swing-out

The relatively high crossover speed for the RSB1 bicycle leads one to contemplate the possibility of balancing the bike at slow speeds, *below* the crossover. Returning to Fig 15, one can see that low speed control which leverages the “swing-out” acceleration would be a difficult. The lowest speed for which the author was able to ride EZRSB had an estimated balance authority of approximately *b*_*u*_ = 0.06. According to the plot in Fig 15, the largest magnitude of *b*_*u*_ that one could experience while traveling slower than the crossover speed is estimated to be |*b*_*u*_| = 0.05. This occurs at half the crossover speed, giving a pole-to-zero ratio of *p*/*z* = 2, about half that recommended by Åström [12] for robustness.

Furthermore, to ride a rear-steered bicycle and keep it balanced at these low speeds, where the swing-out component of acceleration dominates, requires a fundamentally different approach to riding the bike. The physics of the system, combined with the controllability analysis suggests the following strategy:

1. *Speed control.* The reason why the balance authority curve for RSB1 takes the shape shown in Fig 15 is because of the swing-out acceleration is linearly proportional to speed and the centripetal part of lateral acceleration is quadratic. For the rear-steered bike, these two components oppose each other, albeit 90° out of phase. The figure shows that balance authority is quite small for all speeds slower than the crossover. But if such an approach is to work, then it would be important for the rider to get into and remain within a narrow range of speeds near $\frac{1}{2}v_{cr}$ where the magnitude of *b*_*u*_ is largest.
  It is critically important that the rider develop a “feel” for how the bicycle is responding to their control inputs. If the rider recognizes that the bike is not responding sufficiently well, then the rider should know how to speed up or slow down to improve the response.
2. *Exploit the “swing-out.”* For low speeds, the lateral component of acceleration that has the strongest impact on lean dynamics is the “swing-out” component. The rider must re-train their brain to recognize that it is not the steer angle *δ* that is used to balance the bike at low speed. Instead, it is the rate at which one turns the handlebar, $\dot{\delta }$, to swing the rear wheel outward, that is important. *The rider must always be thinking of generating* $\dot{\delta }$, *not δ as a way to balance the bike.* Fortunately, the swing-out acceleration is something the rider can *feel*. The rider will *not* feel it in their arms, or in their feet. The rider will feel it through their butt attached to the seat. This is because the seat on RSB1 is mounted directly above the steered wheel, where the swing-out acceleration is greatest. One can deliberately connect that feel to the $\dot{\delta }$ steer rate input. Then, the strategy becomes a task of leveraging the swing-out acceleration in effort to combat bicycle lean. To the author, this is why developing a simple intuitive understanding of balancing physics of the rear-steered bike is important. The knowledge guides the process of learning to ride RSB1.
3. *Configure the steer kinematics.* Since the control strategy is to consciously prioritize swinging the bicycle’s rear end into the direction of a lean, rather than steering the direction of the bike, the author believes it is important to configure the steer chain as a simple loop rather than a figure eight as shown in Fig 2. This way, the rear end of the bike accelerates into the direction that the turned handlebar on RSB1 is turning. This is the opposite of what one should choose in the “Ride Fast” strategy.
4. *Direction control.* An important part of Klein’s Rear-Steered Bicycle Challenge is to be able to control the direction of the bike. It is not sufficient just to keep it balanced. Klein claimed that at least one rider was able to achieve balance, but “the rider ended up riding haphazardly in an open and flat parking lot as opposed to being able to follow a prescribed path.” [10].
  As stated in item 2, the primary means of affecting the lean dynamics of the bike is through $\dot{\delta }$, the rate at which the rider turns the handlebar, not the handlebar angle itself. Once the rider becomes proficient at keeping the bicycle balanced, They may learn to coordinate the $\dot{\delta }$ balance inputs to produce a non-zero average steer angle *δ* which steers the bike in the desired direction. It just takes practice.

## Results (ride slowly and exploit the swing-out)

The author’s students designed and built a rear-steered bicycle similar to Klein’s Rear-Steered Bike I. After about a month of training to ride slowly and to exploit the swing-out on this rear-steered bike, the author was able to properly control speed, to reliably keep the bike balanced, and to maintain directional control. A few months later, the author coordinated a visit with Richard Klein, and demonstrated that he was able to implement the ride slowly and swing-out strategy on Klein’s Rear-Steered Bike I as well, satisfying the conditions specified in the challenge.

Within weeks, one of the author’s students, Joe Szalko, also demonstrated that he was able to ride RSB1.

S5 Video shows a video in which the author successfully rides Rear-Steered Bike I (RSB1). The video shows a typical ride on RSB1. In comparison to the EZRSB (the “Easy Rear-Steered Bike”) of S3 Video, riding RSB1 is a struggle. It demands the rider’s complete attention. The rider consistently provides large steering input to keep the bike balanced. And the speed of the bike is slow, equivalent to a leisurely walking pace of 15.5 minutes per kilometer (25 minutes per mile). This is the nature of riding the previously “unridable” bicycle.

Videos recorded on multiple occasions over separate days show that the rider consistently chooses to ride the bike at a speed very close to 1.1 m/s (2.4 mph). This consistency suggests that the rider is able to achieve the speed control component (Part 1) of the “Ride Slow” strategy. It is worth noting that the rider’s speed of 1.1 m/s closely matches the bike speed that maximizes the magnitude of balance authority predicted in Fig 15.

Even so, that maximal balance authority at $v\approx \frac{1}{2}v_{cr}$ is still very small. This is why S5 Video shows the rider continually making rapid steer angle changes. Occasionally, one sees steering angle changes of more than 60° or more over a small fraction of a second. The RSB1 rider needs to be vigilant and aggressive at all times.

In the linearized model (13) adopted for control analysis, there is no limit to the size of the control input *u* that one could apply, or the amount of time that one could apply it. Here, in the experiment, one sees that there are limits to how much the rider can actually turn the handlebar. In multiple occasions in the video, the rider is near the limit.

There are occasions in the video where the rider deviates from item 2 of the “Ride Slow” strategy. These occur when the bike is in a very tight turn. In this case, centripetal acceleration dominates, and one can maintain the tight turn by making small handlebar input and even by modulating one’s speed. It is a type of riding that does not seem to have a front-steer counterpart. It is rather fun.

S6 Video shows a rider on RSB1 again, starting at the normal speed of approximately $\frac{1}{2}v_{cr}$. The rider then increases speed and attempts to ride as fast as possible without losing balance. Repeated measures indicate that this occurs at about 1.6 m/s. This corresponds to *b*_*u*_ = 0.04 and *p*/*z* = 1.3. Performing the same experiment, but this time testing how slowly the bike can travel without losing the ability to balance and maintain directional control, reveals a lower bound on the ridable speed at approximately 0.7 m/s, with *b*_*u*_ = 0.04 and *p*/*z* = 3.0.

Thus, one can conclude that it is possible to ride Klein’s Rear-Steered Bike I, maintaining balance and directional control, within a narrow range of slow speeds. Riding the bike within this range requires one to adopt an unconventional strategy that takes advantage of a “swing-out” component of acceleration that depends on the rate at which the rider turns the handlebar. Puzzle solved. Challenge completed.

## Conclusion

When a five-year-old child learns to ride a bicycle, she does not perform centripetal acceleration calculations, plot wheel trajectories, and then conduct state space stabilizability analysis. Instead she hops onto the saddle, propels herself forward, falls, gets back up, and tries again, and again, and again. The self-learning neural network inside her brain builds models of how the bike works, and sifts through promising control strategies. In the end, she develops an ability to ride a bike.

There is no reason to believe that a human, with a sufficient amount of experience and exploration with the rear-steered bike, could not form the appropriate neural connections that would lead to an ability to ride Klein’s “Unridable” Bicycle, without ever contemplating a differential equation.

But there is a reason Klein and his collaborators deemed his rear-steered bicycle “unridable.” It is because the bike exhibits a non-minimum phase behavior in which the open-loop zero is in close proximity to an unstable pole. This article characterizes it as a condition of marginal controllability, unforgiving to lapses in concentration, while working only within a narrow range of speeds.

The exploration described here is the first to characterize the difficult-to-control dynamics, physically, as a consequence of two types of counteracting lateral accelerations that a rider can *feel*, and that a rider can *generate* through the steer angle, and the rate of change of the steer angle. A state space perspective illuminates a geometric understanding of what happens at a particular “crossover speed” when controllability is lost. At bike speeds faster than the crossover, riding the rear-steered bike is similar to riding a traditional bicycle: one steers into the direction of the lean. However, getting the bike past the crossover speed is a formidable task. Instead, by developing a measure of balance authority, one can identify a narrow range of bike speeds below the crossover where stabilization seems theoretically possible. It requires an unorthodox approach to balancing the bike. It’s not *how much* one turns the handlebar that is particularly important to keep the bike upright, but rather it is *how fast* one turns the handlebar that matters most, even if the handlebar is turned the “wrong way.” The author feels that understanding the physics of the system greatly helps in teaching oneself how to ride a bike using this alternate strategy.

The primary contribution of the paper is a demonstration that Klein’s “unridable” rear-steered bike is ridable. Video footage of the feat, provided in the supporting information (S5 Video), shows that riding the bike is inelegant. It is a jarring and frenetic sequence of over-corrections and under-corrections that looks more like a nervous breakdown than a technical or athletic triumph. But it is an accomplishment of a task that was deemed impossible by scholars with enough confidence in their claim that they offered a nontrivial cash prize. The puzzle is solved.

Klein [1] and Åström et al [2] used the “unridable” rear-steered bicycle as a curious and cautionary case study suitable for engineering students studying dynamic systems and control. In much the same way, this study, which takes a deeper exploration into the modeling and successful, albeit delicate, control of the system might be a valuable case study for advanced undergraduates and beginning graduate students in engineering.

## Supporting information

S1 Video Students’ attempts to ride Rear-Steered Bike I. Typical attempts to ride a rear-steered bicycle. Attempts appear to be similar regardless of whether the steer chain is configured as a simple loop or figure-8 as depicted in Fig 2. Students in the video are adults and have provided written consent.
(MP4)

S2 Video Attempt at riding the easy rear-steered bike (EZRSB), but without sufficient initial speed. The author’s push off from a lamppost was not sufficient to propel the bike and rider sufficiently past the crossover speed at which the bicycle could be balanced easily.
(MOV)

S3 Video A successful ride of EZRSB. With sufficient initial speed, the author demonstrates that EZRSB can be balanced with little effort. The end of the video shows that coming to a stop is a different matter.
(MP4)

S4 Video Riding EZRSB at a slow speed close to the balanceability boundary. At a speed of *v* = 1.1 m/s, it becomes difficult for the author to maintain balance and directional control.
(MP4)

S5 Video Success at riding Klein’s “unridable” Rear-Steered Bike I. Video of the author successfully riding RSB1.
(MP4)

S6 VideoRear-Steered Bike I traveling (relatively) fast.The bike is ridden at its normal speed close to $\frac{1}{2}v_{cr}$. Then speed is increased until the rider (the author) can no longer balance it. During this, the rider is attempting to travel in a straight line. The precursor to losing balance is a loss of directional control.(MP4)

S1 Appendix Validation model for rear-steered bike, details for comparison. Notes that show how the validation model for the rear-steered bicycle was obtained from (2) and how coefficients contain elements that can be removed for comparison to the simplified model.
(PDF)
