# Supplementary material for: The right way to ride the wrong bike: An exploration of Klein’s ‘unridable’ bicycle
Source: PLoS One. 2025 Jan 2;20(1):e0315769. doi: 10.1371/journal.pone.0315769 (PMC11695032; doi:10.1371/journal.pone.0315769)
Supplement: S1 Appendix — Notes that show how the validation model for the rear-steered bicycle was obtained from (2) and how coefficients contain elements that can be removed for comparison to the simplified model. (PDF) [file pone.0315769.s007.pdf]

S7 Appendix: Validation Model for Rear-Steered Bike and Suitability of Assumptions

The paper shows how one can obtain a validation model, derived from the "benchmark" model of Meijaard, Papadopoulos, Ruina, and Schwab. [1] (subsequently referred to as MPRS), that is appropriate for steer angle input rather than steer torque input. In this appendix, we show how one can re-frame, the equations of motion so that they represent the dynamics of the *rear*-steered bicycle with coordinate directions defined in the current study. The Validation Model (Eq (2) in the accompanying article), obtained by direct reduction the benchmark model, is reproduced here for the reader's convenience:

$$M_{\theta\theta}\,\ddot{\theta} + g\,K_{0\theta\theta}\,\theta = -M_{\theta\delta}\,\ddot{\delta} - g\,K_{0\theta\delta}\,\delta - v\,C_{1\theta\delta}\,\dot{\delta} - v^2\,K_{2\theta\delta}\,\delta. \tag{1}$$

Except for the fact that we use θ to indicate the lean angle of the bike, whereas MPRS. use ϕ, the names of the dynamic variables and coefficients are the same. The original derivation is valid regardless of whether parameter v is positive or negative. Therefore the model can be used for studying the dynamics of front-steered and rear-steered bicycles. Also, in the original derivation, the "positive" vertical direction is pointed downward as indicated by the downward-pointing $\hat{\boldsymbol{e}}_3$ in Fig 1A

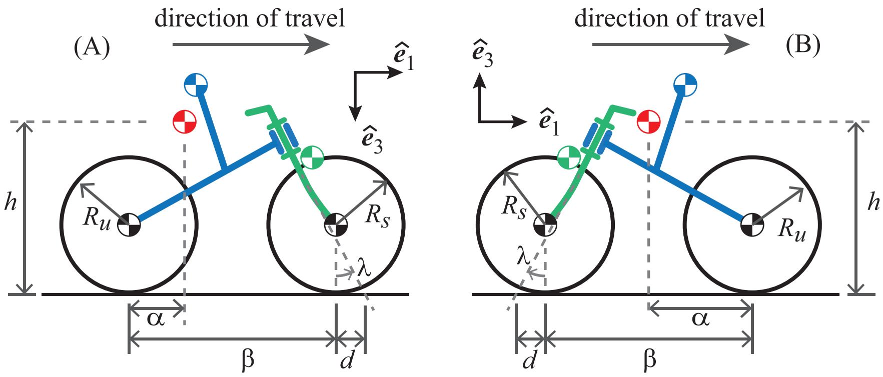

Fig 1. Carvallo-Whipple bicycle model used by Meijaard et al. [1] to derive equations of motion. (A) Front-steered bicycle. (B) Rear-steered bicycle.

The purpose of this appendix is to compare the validation model to the simplified model of the rear-steered bicycle derived in the accompanying paper. Mathematically, the simplified model can be represented by a second order differential equation below with steering input δ in Eq (13) of the paper. It is reproduced here for the reader's convenience:

$$I_{c1}\ddot{\theta} - mgh\,\theta = mh\left(\frac{v^2}{\beta}\delta - \frac{\alpha v}{\beta}\dot{\delta}\right). \tag{2}$$

Mechanically, the simplified model of the rear-steered bicycle consists of an inverted pendulum attached to a carriage shown in Fig 2.

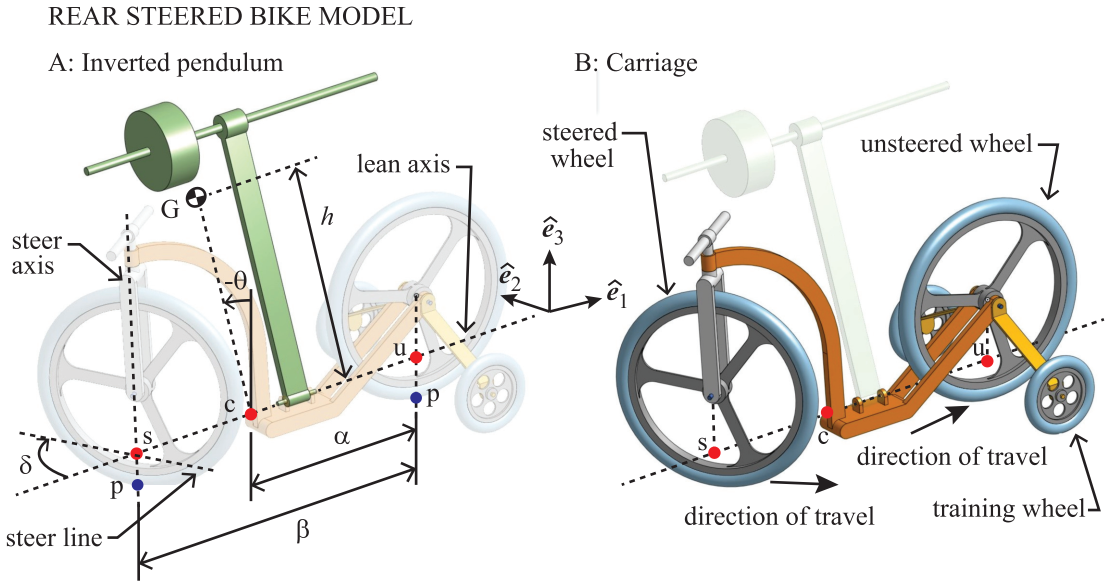

Fig 2. Simplified mechanical model of the rear-steered bicycle. The model consists of a pendulum attached to a carriage.

The pendulum-carriage model of the bicycle can be thought of as a way of mechanically implementing four simplifying assumptions of the bicycle's dynamics. The assumptions are:

- **Assumption A1: Gyroscopic effects of the rotating wheels are small and can be ignored.** By separating the spinning wheels from the pendulum in the mechanical model, the wheels do not lean when the pendulum does. Therefore gyroscopic moments do not get transmitted back to the pendulum.
- **Assumption A2: Caster effects and the consequences of a tilted steer axis are small and can be ignored.** In the mechanical model, the steer axis is vertical and the contact point between the steered wheel and the ground lies on the steer axis.
- **Assumption A3: The offset of the center of mass of the steer assembly from the steer axis has a small effect that can be ignored.** The center of mass of the steer assembly for the mechanical model is assumed to be on the steer axis.
- **Assumption A4: Efforts to resolve fine details of the mass distribution of the bike/rider has a small effect and can be ignored.** In the mechanical model, the lean dynamics are represented by a simple pendulum with two parameters: h is the distance of the center of mass from the lean axis; I_{c1} is the moment of inertia of the pendulum about the lean axis.

This appendix addresses two main questions:

1. If one applies the same assumptions A1 through A4 to the MPRS Validation Model in Eq (1), does one obtain equations of motion equivalent to those of the simplified model of Eq (2)?

2. Do the assumptions A1 through A4 represent quantitatively small effects that can be ignored in studying the non-minimum phase behavior of a rear-steered bicycle and its effect on controllability/stabilizability?

Comparison of Validation Model to Simplified Model

Any attempt to compare the rear-steered bike Simplified Model to that of the Validation Model must reconcile the sign conventions. To re-cast Eq 1 into a form which is compatible for direct comparison to Eq 2, one must make the following mappings:

$$(\hat{\boldsymbol{e}}_1, \hat{\boldsymbol{e}}_2, \hat{\boldsymbol{e}}_3) \mapsto (-\hat{\boldsymbol{e}}_1, -\hat{\boldsymbol{e}}_2, -\hat{\boldsymbol{e}}_3), \qquad v \mapsto -v, \qquad \theta \mapsto -\theta, \qquad \delta \mapsto \delta, \tag{3}$$

and then multiply both sides of the resulting equation by -1 to get comparable signs. The resulting Validation Model for the rear-steered bike becomes

$$I_{c1}\ddot{\theta} - mgh\,\theta = M_\delta\,\ddot{\delta} - g\,K_0\,\delta - v\,C_1\,\dot{\delta} + v^2\,K_2\,\delta. \tag{4}$$

Pendulum Terms are Identical

We refer to the two terms involving $\ddot{\theta}$ and θ on the left side of Eq 4 as the (linearized) "pendulum" terms since these are the ones that appear by virtue of the fact that the bike behaves like an inverted pendulum. The terms on the right side have the steer input (δ, $\dot{\delta}$, $\ddot{\delta}$) and represent forcing terms that the rider applies to attempt to balance the inverted pendulum.

When one applies the mappings (3) to Eq (1) and deciphers the result of this action on the MPRS coefficients $M_{\theta\theta}$ and $K_{0\theta\theta}$, one finds that the pendulum terms in the validation model (4) are identical to those of the simplified model (2), without applying any of the simplifying assumptions A1 through A4.

Here, m is the total mass of the bike with rider (m_T in MPRS), h is the distance of the center of mass of the total bike with rider from the pivot contact with the ground ($-z_T$ in MPRS). The coefficient I_{c1} is the moment of inertia about the ground point of contact along the longitudinal $\hat{\boldsymbol{e}}_1$ direction. MPRS call this term I_{Txx} which they obtain by applying the parallel axis theorem to all four bodies of the Whipple-Carvallo model of the bike, Fig 1.

The first two data columns of Table 1 shows values of coefficients I_{c_1} and $m\,h$ corresponding to the parameters of the MPRS "benchmark" bicycle. For each row, the pendulum coefficients have the same value because they are not affected by the assumptions.

Table 1. Coefficients for the Validation Model (4)**.** Numeric values come from parameters published by Meijaard et al. [1], with some modifications to better match Klein's rear-steered bike, RSB1. Differences from MPRS: $\beta = 1.09\,\text{m}$, $\alpha = 0.61\,\beta$, $d = 0.025\,\text{m}$.

**Assumptions**	I_{c1} (kg m^2)	mh (kg m^2/s^2)	M_δ (kg m^2)	K_0 (kg m)	C_1 (kg m)	K_2 (kg)
None	80.82	80.95	1.384	1.564	51.48	71.68
A4 Only	80.82	80.95	1.285	1.564	51.48	71.68
A3, & A4	80.82	80.95	1.174	1.364	51.49	71.68
A2, A3, & A4	80.82	80.95	0	0	50.19	75.37
All	80.82	80.95	0	0	49.39	74.27
All, Symbolic	I_{c_1}	mh			$m\,h\,\alpha/\beta$	$m\,h/\beta$

Coefficient M_δ

After re-mapping and transcribing the $M_{\theta\delta}$ coefficient from MPRS, first coefficient on the right side can be written as

$$M_\delta = m_A\, u_A h_A + I_{A_{11}} \sin(\lambda) + I_{A_{13}} \cos(\lambda) + \mu I_{U_{13}}. \tag{5}$$

This is an inertial term. Quantities m_A, h_A, and I_A represent the combined mass, center of mass height, and various moments and products of inertia of the steer assembly (fork and handlebar), along with the steered wheel. We will call this the steered mass.

- The quantity u_A in the first term is one which MPRS define as a measure of how far the center of mass of the steered mass is away from the steer axis. It is an effect that vanishes when Assumption A3 is applied.
- The next two terms in Eq (5) provide a measure of how well the principal axes of inertia of the steered mass align with the steered axis. Under Assumption A4 these two terms would go to zero.
- The last term in Eq (5) contains the quantity $\mu = (d/\beta)\cos(\lambda)$. Recall from Fig 1 that the quantity d is the mechanical trail, or caster length. Under Assumption A2, such caster effects vanish. The quantity $I_{U_{13}}$ will be discussed further in the examination of coefficient C_1. It also has some parts that vanish when Assumption A4 is enforced.

For reasons detailed above, **the coefficient M_δ is *zero* under the assumptions of the Simplified Model.** Table 1 shows non-zero values of M_δ when only some of the assumptions are applied, including when no assumptions are applied. Note that the value of this coefficient, without assumptions, is different from the value published in Meijaard et al. [1] since we are using values of the mechanical trail, d, and wheel base, β, and $I_{T_{13}}$ that are different from those of the benchmark bicycle, and better represent RSB1.

Coefficient K_0

The term containing coefficient K_0 in Eq (4) is called the static moment. It is given by

$$K_0 = -m_A\, u_A - \mu\, m\, \alpha. \tag{6}$$

Observations:

- If the center of gravity of the steered mass does not lie on the steer axis (Assumption A3 violated), then turning the handlebar causes the some of the bike's weight to shift laterally, altering the gravitational torque acting on the bike. This is the effect of the first term in Eq (6). which contains the center of steered mass offset, u_A.
- Similarly, the second term contains the caster ratio $\mu = (d/\beta)\cos(\lambda)$ which vanishes with the application Assumption A2. When a bike has caster, turning the handlebar causes the center of mass of the frame and rider to shift laterally, also causing a change in the gravitational moment.

As described above, **the coefficient K_0 is *zero* under the assumptions of the Simplified Model.** Values of the coefficient are displayed in Table 1 when assumptions are removed. We note again that these values are different from those of the MPRS model because of geometric differences and differences in center of mass location on a rear steered bike, parameter α.

Coefficient C_1

The term containing coefficient C_1 in Eq (4) is the one which accounts for the "swing-out" acceleration in the Simplified Model. Below we see that the corresponding term in the MPRS model contains additional effects.

$$C_1 = \mu \left(\frac{I_{u2}}{R_u} + \frac{I_{s2}}{R_s} + m\,h \right) + \frac{I_{s2}}{R_s} \cos(\lambda) + I_{U_{13}} \frac{\cos(\lambda)}{\beta} \tag{7}$$

Observations:

- The quantities I_{u2} and I_{s2} are moments of inertia of the unsteered and steered wheels, respectively, about the spin axis, R_u and R_s are their corresponding radii. Terms with these moments of inertia are the ones which generate the gyroscopic effects that Assumption A1 seeks to eliminate.
- As before, the quantity μ is a caster effect that goes to zero when Assumption A2 is activated.
- There are instances in Eq (7) that have a $\cos(\lambda)$ multiplier. These do not directly correspond to a caster effect. However, in keeping with the vertical steer axis stipulation in Assumption A2, one should replace $\cos(\lambda)$ with 1, when the assumption goes into effect.
- Finally, there is a term containing the quantity $I_{U_{13}}$. (MPRS calls it $I_{T_{xz}}$.) It is a product of inertia, based at the location where the unsteered wheel makes contact with the ground, characterizing the imbalance of mass with respect to the $\hat{\boldsymbol{e}}_1$ and $\hat{\boldsymbol{e}}_3$ directions. MPRS determine this quantity by applying the parallel axis theorem, taking into account the mass distribution of the frame/rider, the steer assembly, and the steered wheel.

  When applying Assumption A4, though, we model the mass distribution of the whole bike as a simple pendulum situated a horizontal distance α from the ground contact point of the unsteered wheel, and with a center of mass a distance h from above ground. Therefore, with the application of Assumption A4, The product of inertia becomes $T_{U_{13}} = m\,\alpha\,h$.

Therefore, we find that, under the assumptions of the Simplified Model, the coefficient becomes

$$C_1 = \frac{m\,h\,\alpha}{\beta} \tag{8}$$

Values of coefficient C_1 obtained by substituting parameters for the benchmark bike and RSB1, are shown in Table 1. The bottom numerical row shows the value when all assumptions are applied. The rows above show how the value of the coefficient changes as assumptions are removed. The values for C_1 never fluctuate my more than 5%. This seems to confirm the rationale for the assumptions: they allow us to simplify the model significantly with a relatively small loss of precision.

Coefficient K_2

The term containing coefficient K_2 in Eq (4) is the one which accounts for the centripetal acceleration in the Simplified model. In the MPRS-based Validation Model, it contains additional terms.

$$K_2 = \left(\frac{I_{u2}}{R_u} + \frac{I_{s2}}{R_s} + m\,h \right) \frac{\cos(\lambda)}{\beta}. \tag{9}$$

Observations:

- The terms with the wheel rotational inertias I_{u2} and I_{s2} are used for constructing derivatives of angular momenta. The terms represent gyroscopic effects. Assumption A1 gets rid of such gyroscopic effects.
- When Assumption A2 is activated, the steer axis becomes vertical, meaning $\cos(\lambda) = 1$.

Therefore, we find that, under the assumptions of the Simplified Model, the coefficient becomes

$$K_2 = \frac{m\,h}{\beta} \tag{10}$$

Values of coefficient K_2 obtained by substituting parameters for the benchmark bike and RSB1, are shown in Table 1. The bottom numerical row shows the value when all assumptions are applied. The rows above show how the value of the coefficient changes as assumptions are removed. The values for K_2 never fluctuate by more than 4%. This seems to confirm the rationale for the assumptions: they allow us to simplify the model significantly with a relatively small loss of precision.

Term by Term Comparison

The bottom row of Table 1 shows a symbolic representation of the terms that survive in each of the coefficients of the Validation Model in Eq (4) after applying Assumptions A1 through A4. Terms containing coefficients M_δ and K_0 vanish completely. Terms containing coefficients C_1 and K_2 simplify considerably. Substitutions into Eq (4) yield

$$I_{c1}\ddot{\theta} - mgh\,\theta = -v\frac{mh\alpha}{\beta}\,\dot{\delta} + v^2\frac{mh}{\beta}\,\delta. \tag{11}$$

Comparison with the Simplified Model (2), shows that the equations of motion for the Simplified Model are *exactly the same* as those for the Validation Model when Assumptions A1 through A4 are applied.

It is worth noting that Eq (2) was *not* obtained by first deriving equations of motion of a realistic bicycle, like that of Carvallo and Whipple, and then applying simplifications to the mathematical equations. (This is how we obtained Eq (11).) Instead, (2) was produced by imposing *mechanical* simplifications to the physical bicycle and then deriving equations of motion for the resulting pendulum/carriage. It is interesting to note that these two approaches produced the same result. In this sense, we say that the pendulum/carriage model bike is a mechanical analog of a bicycle, subject to the assumptions.

Role of Assumptions in Response to Steer Input

In the full Validation Model (4) and in the Simplified Model (2, 11), the left side of the equations represent the dynamics of the inverted pendulum, while the right side consists of forcing terms that represent how the rider can effectively generate moments on the bike through a steer input. In the accompanying article, the author showed, with a simple steer input, why the rear-steered bike RSB1 is so difficult to balance. We reproduce the construction here to observe how relaxation of the assumptions might alter the conclusions.

Suppose, for example, that a bicycle is leaning to the left. In order to prevent the bike from falling to the left, the typical response of a cyclist would be to turn the bike to the left, creating a lateral acceleration to the left. If the turn is sufficiently aggressive, a conventional understanding of bike dynamics would expect that the action would upright the bicycle. Fig 3A shows the example steer input considered in the

accompanying article. It depicts how a rider might smoothly increase the steer angle δ from zero to about 30° (solid curve) with a 10% to 90% rise time of 0.5 seconds. The dashed and dotted curves in the figure depict the corresponding plots of $\dot{\delta}$ and $\ddot{\delta}$ respectively.

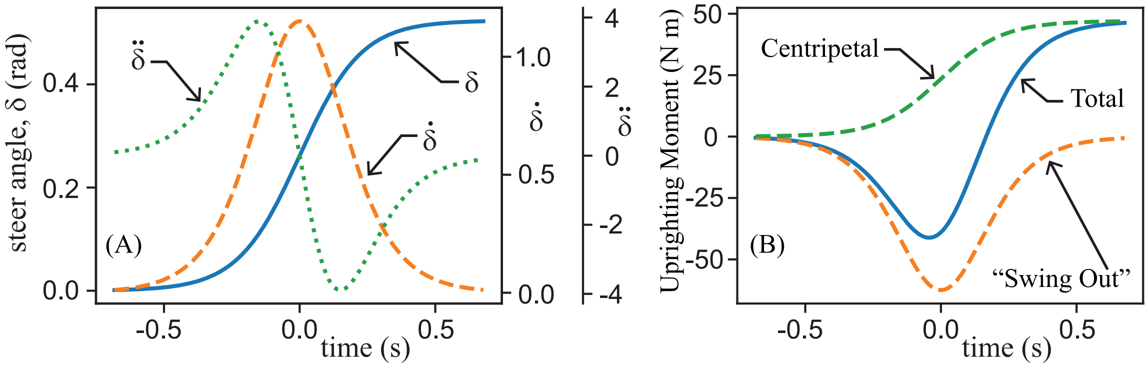

Fig 3. Steer input and its effect on the uprighting moment acting on the Simplified Model of the rear-steered bike. (A) Steer input. Plots of steer angle, δ, and the corresponding time derivatives, $\dot{\delta}$ and $\ddot{\delta}$. (B) A plot of the right side of Eq 11 evaluated with the corresponding steer command.

Fig 3B shows what happens when one substitutes the steer input in Fig 3A into the right side of Eq 11, the Simplified Model with all four assumptions A1 through A4 applied. The result, indicated by the label "Total" is the effective uprighting moment that the rider generates along the lean axis in effort to arrest the leftward falling action of the bike.

For the simplified model of the rear-steered bike, there are just two terms in the function. One is due to centripetal acceleration acting on the bike, as indicated in the figure. The other term provides a competing "swing-out" acceleration effect acting in the opposite direction, just shifted in phase by 90°. The result is a non-minimum phase behavior [3] that causes the steering action to produce a destabilizing response at first before finally settling on a response that could help the rider, if the rider hasn't fallen yet. The time scale associated with this non-minimum phase response, relative to the falling time scale of the bike made it nearly impossible to ride Klein's RSB1.

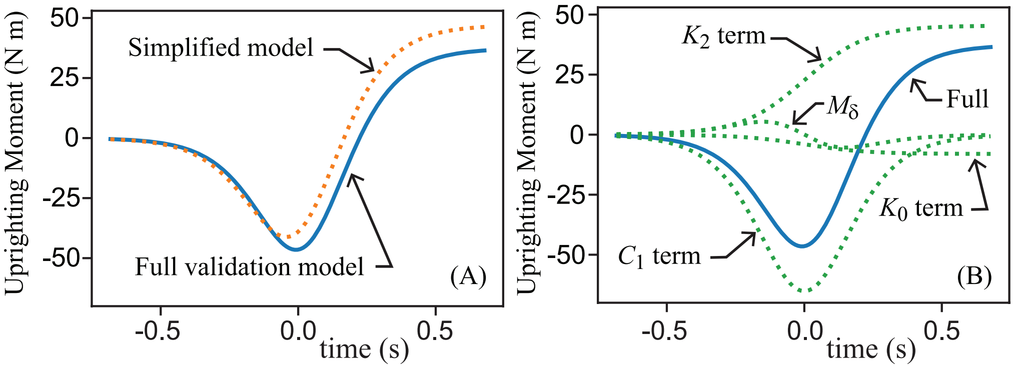

Fig 4. Uprighting moment calculated by incorporating all four terms in the Validation Model (4) **of the rear-steered bike.** (A) Comparison to simplified model with all four assumptions applied. (B) Illustrating the contributions of each of the four terms on the right side of the "full" Validation Model.

In Fig 4A, we show the uprighting moment response to the same steer input. But this time, all four terms on the right side of the Validation Model (4) are included in the calculation. None of the Assumptions A1 through A4 are applied. These plots illustrate, like the coefficients in Table 1, that the terms removed by the assumptions are quite small compared to the centripetal and "swing out" acceleration effects that the Simplified Model focuses on. The non-minimum phase behavior persists both quantitatively and qualitatively. Both responses to the steer input exhibit similar timescales at which the plot switches sign, indicating that they have similar controllability difficulties, with small modifications to quantitative details.

Fig 4B shows how each of the four terms on the right side of Eq (4) contribute to the pendulum forcing behavior. It is a small contribution. The author is justified in focusing on the effects of centripetal and swing-out acceleration in learning how to balance the rear-steered bike.

References

1. Meijaard JP, Papadopoulos JM, Ruina A, Schwab AL. Linearized dynamics equations for the balance and steer of a bicycle: a benchmark and review. Proceedings of the Royal society A: mathematical, physical and engineering sciences. 2007;463(2084):1955–1982.

2. Kooijman JDG, Meijaard JP, Papadopoulos JM, Ruina A, Schwab AL. A Bicycle Can Be Self-Stable Without Gyroscopic or Caster Effects. Science. 2011;332(6027):339–342. doi:10.1126/science.1201959.

3. Åström KJ, Murray RM. Feedback Systems: An Introduction for Scientists and Engineers. Princeton University Press; 2008.
